# Comparison of phenolic profiles and antioxidant activities in the pulps from 21 different *Artocarpus heterophyllus* Lam. cultivars

**DOI:** 10.1016/j.fochx.2025.102735

**Published:** 2025-07-04

**Authors:** Ming Cheng, Mengyang Liu, Lehe Tan, Chuan Li, Gang Wu, Bingqiang Xu, Yanjun Zhang, Kexue Zhu

**Affiliations:** a State Key Laboratory of Tropical Crop Breeding, Spice and Beverage Research Institute, Chinese Academy of Tropical Agricultural Sciences, Sanya/Wanning Hainan 572024/571533, China; b School of Food Science and Engineering, Hainan University, Haikou, Hainan 570228, China; c School of Food Science and Engineering, Ocean University of China, Qingdao, Shandong 266003, China; d Sanya Research Institute of Chinese Academy of Tropical Agricultural Sciences, Chinese Academy of Tropical Agricultural Sciences, Sanya, Hainan 572024, China; e Key Laboratory of Processing Suitability and Quality Control of the Special Tropical Crops of Hainan Province, Wanning, Hainan 571533, China; f National Center of Important Tropical Crops Engineering and Technology Research, Wanning, Hainan 571533, China

**Keywords:** *Artocarpus heterophyllus* Lam., Phenolic profile, Antioxidant activity, UPLC-ESI-Q-TOF-MS/MS

## Abstract

*Artocarpus heterophyllus* Lam. (jackfruit) exhibits important biological activities, with its antioxidant effects are closely associated with rich phenolic compounds. This study evaluated the total phenolic content (TPC), total flavonoid content (TFC), and phenolic components in jackfruit pulp from 21 different cultivars. 58 phenolic compounds were identified with significant variations in their quantity and content among the cultivars by using UPLC-ESI-Q-TOF-MS/MS. Notably, the cultivar “Malaysia 1” had the highest TPC and TFC, exhibited the strongest antioxidant capacity. A strong correlation between polyphenol content and antioxidant properties suggests that procyanidins B1 and *p*-coumaric acid are key contributors to the antioxidant activity of jackfruit pulp. Principal component analysis (PCA) revealed distinct patterns in the phenolic compound profiles across samples. These findings provide a theoretical basis for the precise cultivation of high-antioxidant varieties possible and provide a reference for the processing strategies of targeted functional jackfruit products.

## Highlights

• A total of 66 phenolics were identified in jackfruit pulp from different cultivars.
• Positive correlation existed between antioxidant activity and phenolic content.
• Malaysia1, was proven to exhibit the most intensive antioxidant capacity.
• Procyanidin B1 and *p*-coumaric acid may be the main phenolic compounds affecting the antioxidant activity of jackfruit pulp.

## 1 Introduction

*Artocarpus heterophyllus* Lam. (jackfruit), belonging to the Moraceae (Mulberry) family, produces the largest edible fruits. Globally, jackfruit plantations cover an area exceeding 300,000 hectares, with an annual yield of approximately 3.7 million tons (Spada et al., 2021). Its production has exhibited an annual growth rate of 15%, and the economic value of jackfruit continues to rise in parallel with its expanding cultivation area (Zhang et al., 2018). Jackfruit pulp is a rich source of essential nutrients, including carbohydrates, polyphenols, proteins, essential fatty acids, vitamins, and minerals (Shafiq et al., 2017; Zhu et al., 2021). Moreover, jackfruit exhibits antibacterial, anti-inflammatory, immuno-modulatory, and other pharmacological properties, largely attributed to its polyphenolic compounds (Baliga et al., 2011).

Polyphenols, characterized by hydroxylated phenyl groups, are diverse groups of plant secondary metabolites with well-documented health-promoting properties (Cao et al., 2021). In recent years, the biological activities of polyphenol have garnered significant attention in the fields of food science and bio-medicine. Jagtap et al. (2010) quantified total phenolic content (TPC) and total flavonoids content (TFC) of jackfruit pulp from western Ghats India, reporting values of 0.46±0.014 mg GAE/g and 1.20±0.020 mg RE/g under optimum solvent extraction. They also demonstrated strong radical-scavenging activities using 1,1-diphenyl-2-picrylhydrazyl (DPPH), ferric reducing power assays and N, N-dimethyl-p-phenylendiamine (DMPD) radical cation decolorization assay. Zhang, Gao, et al. (2021) investigated the non-extractable polyphenols (NEPP) in dietary fiber from jackfruit pulp (JDF), focusing on their concentration, composition, and antioxidant properties after treating with acid, alkaline, and enzymatic hydrolysis. Their study revealed that alkaline hydrolysis extracts possessed the highest NEPP content (64.9 mg GAE/10 g DM), most diverse composition (thirty-one compounds), and strongest antioxidant activity (2,2′-azinobis(3-ethylbenzothiazoline-6-sulfonic acid) radical cation scavenging ability (ABTS), Oxygen Radical Absorbance Capacity (ORAC) and Peroxyl Radical Scavenging Capacity (PSC) assays). Although these studies have emphasized the potential health benefits of jackfruit polyphenols, they have examined only one variety or a limited number of phenolic constituents, and a systematic elucidation of metabolic diversity across the extensive jackfruit germplasm remains lacking.

The remarkable genetic heterogeneity observed in jackfruit (*Artocarpus heterophyllus*) stems from millennia of seed-based propagation, resulting in substantial phenotypic variation across cultivation regions including India, Bangladesh, Malaysia, and southern China. This diversity manifests in distinct fruit characteristics such as size variability, pericarp pigmentation, flesh textural properties, and differential polyphenolic composition. Our experimental design intentionally encompasses this genetic spectrum by incorporating both widely cultivated Chinese varieties (e.g., XingLongXiangMi) and introduced Malaysian cultivars (Malaysia 1-7 series). This strategic selection of diverse jackfruit germplasm constitutes a methodological improvement that addresses the limited genetic representation in previous studies. The phenolic profiles of pulp from 21 representative jackfruit cultivars by employing ultra-performance liquid chromatography coupled with electrospray ionization, followed by quadrupole time-of-flight tandem mass spectrometry (UPLC-ESI-Q-TOF-MS/MS) and to quantify key phenolic constituents with ultra-high-performance liquid chromatography coupled with diode-array detector (UPLC-DAD). By elucidating the diversity of polyphenols and their antioxidant capacities across these cultivars, the findings would provide deeper insights into the nutritional diversity of jackfruit and lay a foundation for breeding strategies to enhance its nutritional and functional qualities.

## 2 Materials and methods

### 2.1 Chemicals and reagents

The phenolic standard compounds including gallic acid, caffeic acid, protocatechuic acid, chlorogenic acid, neochlorogenic acid, (+)-catechin, procyanidin B1, procyanidin B2, rutin, (-)-epicatechin, *p*-coumaric acid, ferulic acid, quercitrin, quercetin, phlorizin and phloretin were purchased from Sichuan Weikeqi Biotech Co., Ltd. (Sichuan, China). 1,1-Diphenyl-2-picrylhydrazyl (DPPH), 2,2'-azino-bis (3-ethylbenzothiazoline-6-sulfonic acid) (ABTS) and formic acid used for LC-MS were supplied by Sigma-Aldrich Inc. (St. Louis, MO., USA). HPLC grade acetonitrile was supplied by Merck (Darmstadt, Germany). Ethanol and other chemicals were of analytical grade.

### 2.2 Plant materials and preparation of extracts

**Fig. 1:**
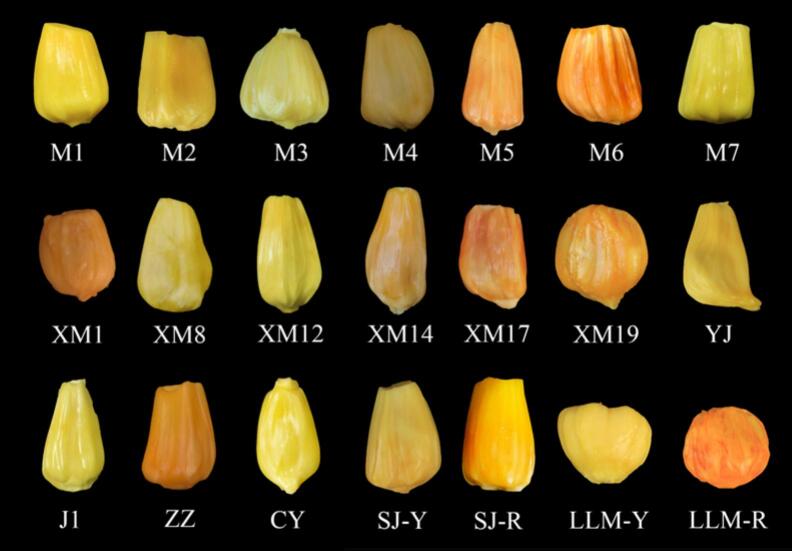
Different cultivars of jackfruit pulp. M1 = Malaysia 1, M2 = Malaysia 2, M3 = Malaysia 3, M4 = Malaysia 4, M5 = Malaysia 5, M6 = Malaysia6, M7 = Malaysia 7, XM1 = Xiangmi 1, XM8 = Xiangmi 8, XM12 = Xiangmi 12, XM14 = Xiangmi 14, XM17 = Xiangmi 17, XM19 = Xiangmi 19, YJ = Yangjiang, J1 = Jiang1, ZZ = Zhenzhuguo, CY = Changyou, SJ-Y = Sijihuang, SJ-R = Sijihong, LLM-Y = Liulianmi-Y, LLM-R = Liulianmi-R.

The mature fruits from 21 jackfruit cultivars (Fig. 1) used in the present study were provided by the Spice and Beverage Research Institute, CATAS. For each cultivar, fruit samples were collected from five distinct, healthy plants representative of the cultivar's typical phenotype within the repository. All cultivars were botanically authenticated, and their developmental stage was confirmed to be at optimal commercial maturity, by Professor Gang Wu (an expert in plant resources science). The determination of maturity was based on a consistent set of standardized horticultural indices, including skin color transition from green to yellow-green, flattening of the carpel spikes, development of a characteristic aroma, and a slight yielding to pressure upon palpation.

Upon harvest, fruits were immediately transported to the laboratory. The pulp was manually separated from the peel and seeds and homogenized. These composite pulp samples were then flash-frozen in liquid nitrogen and stored at -80°C for further analysis. The fruit pulps were processed using our established procedures described in Cheng et al. (2023). Briefly, 1 g of fruit pulp sample was crushed and mixed with 30 mL ethanol/water (60:40, v/v). The extractions were carried at 550 W, 30 °C for 165 s using CW-2000 ultrasonic-microwave cooperative extractor/reactor (Xintuo, Shanghai, China). The extract solution was centrifuged at 10,000 rpm for 10 min at 4 °C. The extraction step was repeated twice and combined supernatants for subsequent determination.

### 2.3 Determination of phenolic and flavonoid contents

TPC was determined using a modified Folin-Ciocalteu method (Peña-Vázquez et al., 2022). Gallic acid (GA) was used to generate the calibration curve at 760 nm in a SynergyH1 microplate reader (BioTek, USA), and TPC results were expressed as mg of GA equivalents per 100 grams of fresh fruit pulp (mg GAE/100 g FW).

TFC was assessed with the AlCl_3_ assay at 710 nm (Suo et al., 2022), with the outcomes reported as mg of (+)-catechin equivalents per 100 grams of fresh fruit pulp (mg CE/100 g FW).

### 2.4 Identification of polyphenolic compounds by UPLC-ESI-Q-TOF-MS/MS

Three microliters of each extract sample was utilized for the identification of polyphenolic compounds, following the methodology established in our previous study (Cheng et al., 2023). UPLC separation was conducted on an Agilent 1290 Infinity II liquid chromatograph system with a ZORBAX RRHD Eclipse Plus C18 column (3.0 mm × 150 mm, 1.8 μm, Agilent, CA, USA). And the electrospray ionization mass spectra were obtained in both positive and negative ion modes using a 6530B Q-TOF mass spectrometer. 16 compounds for standards were confirmed by direct comparison of retention time and MS/MS spectra; the remaining features were tentatively annotated by matching accurate mass and fragmentation patterns according to published literature, aided by retention time trends of structurally related analogues. The specific chromatographic and mass spectrometric conditions have been detailed in our previous literature (Cheng et al., 2023).

### 2.5 Quantification by UPLC-DAD analysis

The corresponding phenolic standards were selected based on the qualitative results identified by UPLC-ESI-Q-TOF-MS/MS. Gradient concentrations (1.0, 5.0, 10.0, 20.0, and 50.0 mg/L) of fifteen phenolic standards were prepared to generate standard working solutions for constructing calibration curves. These curves were used for the quantitative analysis of phenolic compounds in the fruit pulp extracts.

Quantitative analysis was performed using a 1290 Infinity II liquid chromatograph system coupled with an HDR-DAD detector (Agilent, CA, USA). Chromatographical separation was carried out on an Agilent Zorbax SB-C18 column with mobile phases consisted of 2% formic acid (phase A) and acetonitrile (phase B). The gradient elution program was set as follows: the initial proportion of mobile phase B was set at 5% and gradually increased to 25% over the first 30 minutes. Then, from the 30th to the 45th minute, the proportion of phase B was further increased linearly from 25% to 40%. From 45th and 51st minutes, the proportion of phase B was gradually reduced from 40% to 5%. And the proportion of phase B was maintained at 5% for 9 minutes, completing the entire 60-minute program. The flow rate was maintained at 0.8 mL/min, the column temperature was set to 40 °C, and the injection volume was 10 μL. Chromatograms of all analyses were monitored at 280 nm by HDR-DAD UV detector (Wang et al., 2023).

### 2.6 Antioxidant capacity assay

#### 2.6.1 DPPH assay

The abilities of the extracts to scavenge DPPH radicals was evaluated according to the method of Acero and Muñoz-Mingarro (2012). 150 μL DPPH solution (dissolved in 80% methanol) was mixed with 150 μL diluted sample extract, equal-volume of 80% methanol used as a blank control. After incubating at room temperature for 30 minutes in the dark, the absorbance of mixtures were measured at 517 nm. Results were presented as μg Trolox equivalents (TE) per 100 g of fresh mass.

#### 2.6.2 ABTS assay

The assay for the scavenging capacity on ABTS radical was determined following the method outlined by Cheng et al. (2020). A 7 mM ABTS stock solution was reacted with an equal volume of 2.45 mM potassium persulfate to generate ABTS^+^. Then 190 μL of the ABTS^+^ solution was combined with 10 μL of the diluted sample. After incubating at room temperature for 6 minutes in the dark, the absorbance of mixture was recorded at 734 nm. Results were reported as μg TE per 100 g of fresh mass.

#### 2.6.3 Ferric reducing antioxidant power (FRAP) assay

The ferric reducing antioxidant power (FRAP) of the samples was determined according to the commercially available assay kits’ instructions (Suzhou Grace Biotechnology Co., Ltd, Suzhou, China). The absorbance of FRAP solution was measured at 590 nm, and the FRAP value was expressed as μmol TE per 100 g of fresh mass.

#### 2.6.4 Antioxidant potency composite index (ACI)

The antioxidant potency composite index (ACI) was calculated to equally weigh the results from the DPPH, ABTS, and FRAP assays, based on the method proposed by Chen et al. (2020). The formula used was: ACI = (sample score/best score) × 100. The average index score from the three assays was used to represent the ACI for each jackfruit pulp sample.

### 2.7 Statistical analysis

The results are presented as mean ± standard deviation (SD). Statistical analysis was performed using one-way ANOVA, followed by Tukey’s post hoc test in SPSS 27.0 (SPSS, Inc., Chicago, IL, USA) to identify significant group differences at a threshold of p<0.05. Pearson's correlation coefficients were calculated to evaluate the relationship between phenolic content and antioxidant capacity. Principal component analysis (PCA) was conducted with GraphPad Prism 9 (San Diego, CA, USA) to generate a ‘biplot’ representation.

## 3 Results

### 3.1 TPC and TFC

**Fig. 2:**
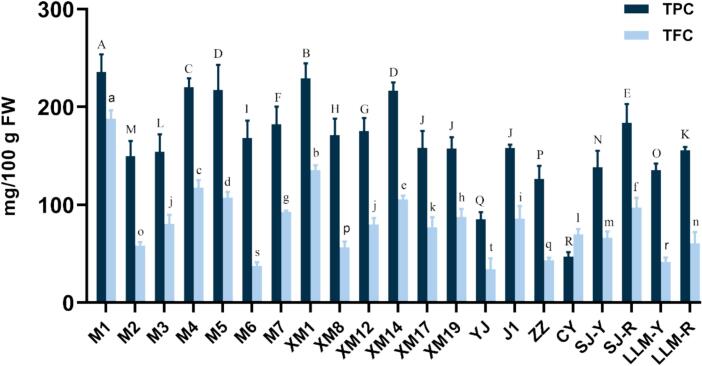
Total phenolic and total flavonoid contents in the pulp of different jackfruit cultivars. Different letters indicate statistically significant differences between cultivars for each variable (*p* < 0.05).

As shown in Fig. 2, the TPC in the pulp of diverse jackfruit cultivars ranged from 46.87 to 235.45 mg GAE/100 g FW, with a mean of 164.95 mg GAE/100 g FW. “M1” (235.45 mg GAE/100g FW), “XM1” (229.16 mg GAE/100g FW), “M4” (220.19 mg GAE/100g FW), “M5” (217.33 mg GAE/100g FW), “XM14” (216.57 mg GAE/100g FW), “SJ-R” (183.77 mg GAE/100g FW), “M7” (182.25 mg GAE/100g FW), “XM12” (175.19 GAE/100 g FW), “XM8” (170.10 GAE/100 g FW) and “M6” (168.14 mg GAE/100g FW) were of 10 cultivars with higher TPC than the average. The TPC varied significantly among the cultivars. “CY” had the lowest TPC (46.87 mg GAE/100 g FW), conversely, “M1” had the highest TPC (235.45 mg GAE/100 g FW), 4.98 times greater than that of “CY”.

The TFC of jackfruit pulp also varied significantly among different cultivars, ranging from 34.11 to 188.01 mg CE/100 g FW, and the mean was 81.96 mg CE/100 g FW. Notably, “M1” had the highest TFC (188.01 mg CE/100 g FW), which was significantly greater than other cultivars, while “YJ” had the lowest (34.11 mg CE/100 g FW). TPC and TFC in jackfruit pulp extracts of all cultivars were much higher than those reported by Jagtapet al. (2010), but the contents in the pulp of “M1” were lower than those determined by Zhang et al. (2017). The above differences could be related to the different preparation methods, varieties and determination methods.

### 3.2 Identification of phenolic compounds

**Fig. 3:**
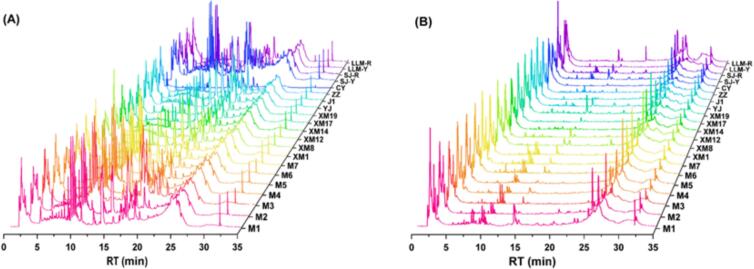
Total ion chromatograms of phenolic compounds in the pulp of different jackfruit cultivars by UPLC-ESI-QTOF-MS/MS in (A) positive ion mode and (B) negative ion mode.

The phenolics and their derivatives in jackfruit pulp extracts from 21 different cultivars were characterized by UPLC-ESI-Q-TOF-MS/MS in both positive and negative ion modes. A total of 58 bioactive compounds were initially characterized by the precise molecular weights of these substances, combined with the mass spectral information and the comparison of the relevant reports, as well as the retention time of the standards under the same conditions. These compounds included 10 flavonols, 9 hydroxycinnamic acids and derivatives, 9 flavanones, 9 flavan-3-ols, 6 hydroxybenzoic acids and derivatives, 6 flavones, 3 coumarins, 2 ellagitannins, 2 chalcones, 1 isoflavone and 1 lignan (Table 1). Total ion chromatograms of the samples were depicted in Fig. 3, and the MS spectra and chemical structures of the compounds were presented in Fig. S1.

**Table 1:** Phenolic profiles of jackfruit pulp from 21 cultivars by UPLC-ESI-Q-TOF-MS/MS.

**Compd**	**RT** **(min)**	**Identification**	**Molecular** **formula**	**Model**	**Parents** **ions**	**Fragment ions**	**Sample**
*Hydroxybenzoic acids and derivatives (6)*							
1	1.75	Bergenin	C_14_H_16_O_9_	+	329.0698	209, 263, 251	SJ-Y, SJ-R, M1, XM19, ZZ, LLM-R
2	3.50	Gallic acid	C_7_H_6_O_5_	+	171.0282	125, 97, 79	SJ-Y, SJ-R, M(1-7), XM(1,8,12,14), YJ, J1, ZZ, CY, LLM-Y
3	4.27	Syringic acid	C_9_H_10_O_5_	-	197.8062	153, 182, 167	SJ-Y, SJ-R, M(1,3,5,7), J1, CY, ZZ, LLM-R
4	6.28	Protocatechuic acid	C_7_H_6_O_4_	-	153.0179	109, 80	M(2,4,6,7), XM(8,12,14,17)
5	8.98	3-O-Galloylquinic acid	C_14_H_16_O_10_	-	343.0837	191	M(1,3,6), XM12, YJ, ZZ, LLM-R
6	10.61	4-Hydroxybenzoic acid	C_7_H_6_O_3_	-	137.0234	93,113, 65	XM12

*Hydroxycinnamic acids and derivatives (9)*							
7	5.44	3-O-Caffeoylshikimic acid	C_16_H_16_O_8_	+	337.0899	179, 173, 135, 191	SJ-Y, SJ-R, M(1-7), XM(1-19), YJ, J1, CY, LLM-Y, LLM-R
8	5.47	5-*p*-coumaroylquinic acid	C_16_H_18_O_8_	-	337.0811	147, 119	SJ-Y, M5, XM19, J1, ZZ, CY, LLM-R
9	6.88	Feruloylputrescine	C_14_H_20_N_2_O_3_	-	263.1385	29, 57, 72	SJ-Y, SJ-R, M(1,2,5,6,7), XM(1-19), YJ, J1, ZZ, LLM-Y
10	7.09	Chlorogenic acid	C_16_H_18_O_9_	-	353.0857	191, 179, 135	SJY, M(3,6,14,17,19), XM19, YJ, LLM-R
11	7.76	Neochlorogenic acid	C_16_H_18_O_9_	-	353.0629	191, 179	SJR, M(1,5), XM(1,14,17), XM19, XL, YJ, J1, CY, ZZ, LLM-Y, LLM-R
12	9.11	*p*-coumaric acid	C_9_H_8_O_3_	-	163.0394	119, 93	SJ-Y, SJ-R, M(1-7), XM(1, 12,14,17), YJ, J1, ZZ, LLM-Y, LLM-R
13	9.25	Methyl rosmarinate	C_19_H_18_O_8_	+	375.1057	179, 135	M(1,4,7), XM1


**Compd**	**RT** **(min)**	**Identification**	**Molecular** **formula**	**Model**	**Parents** **ions**	**Fragment ions**	**Sample**
14	13.52	Ferulic acid	C_10_H_10_O_4_	+	193.0502	163, 135, 43	SJ-Y, SJ-R, M(1-7), XM(1-19), YJ, J1, ZZ, CY, LLM-Y, LLM-R
15	15.89	Caffeic acid	C_9_H_8_O_4_	-	179.0421	135, 79	SJ-Y, XM(8,14), J1, LLM-R

*Flavan-3-ols (9)*							
16	2.13	(-)-Epigallocatechin	C_15_H_14_O_7_	-	305.066	125, 137	XM14
17	2.69	(-)-Epicatechin gallate	C_22_H_18_O_10_	-	441.0846	169, 125, 289	M(2,5,6), XM(12,14,19), CY, ZZ, LLM-R
18	7.56	(+)-Catechin	C_15_H_14_O_6_	+	291.0859	245, 205, 179	SJ-Y, SJ-R, M(1-7), XM(1-19), YJ, J1, ZZ, CY, LLM-Y, LLM-R
19	7.73	(-)-Epicatechin	C_15_H_14_O_6_	+	291.0858	245, 205, 179	SJ-R, XM17, ZZ
20	2.36	Procyanidin B1	C_30_H_26_O_12_	-	577.1562	425, 407, 289	M(1,6), XM(1,14,19), CY, LLM-Y
21	2.68	Procyanidin B2	C_20_H_26_O_12_	-	577.1344	451, 425, 289	XM(8,17,19), CY, LLM-Y
22	1.96	Procyanidin A1	C_30_H_24_O_13_	-	575.1303	539, 407, 285	M4
23	7.15	Procyanidin A2	C_30_H_24_O_12_	-	575.1224	557, 539, 449	M6, XM(8,19)
24	2.54	Procyanidin C1	C_45_H_38_O_18_	-	865.2098	125, 407	LLM-Y

*Flavonols (10)*							
25	1.50	Quercetin	C_15_H_10_O_7_	+	303.0511	179, 151	SJ-Y, SJ-R, M(1-7), XM(1,8,12,17,19), YJ, CY, LLM-Y, LLM-R
26	1.83	(-)-Dihydroquercetin	C_15_H_12_O_7_	-	303.0522	285, 256, 211, 183	M(1,2,3,5,6,7), XM(1,8,17), J1, ZZ
27	5.64	3,7-Di-O-methylquercetin	C_17_H_14_O_7_	+	353.0633	316, 315, 301	SJ-R, M7, XM12


**Compd**	**RT** **(min)**	**Identification**	**Molecular** **formula**	**Model**	**Parents** **ions**	**Fragment ions**	**Sample**
28	8.33	Quercetin-3-O-glucoside	C_21_H_20_O_12_	-	463.0885	301, 300, 271,	M5, XM12, LLM-R
29	2.17	Kaempferitrin	C_27_H_30_O_14_	-	577.1767	285, 193	SJ-Y, SJ-R, M(1,2,5,6), XM(1,8,14,17), J1
30	2.31	Kaempferol	C_15_H_10_O_6_	+	287.0539	257, 229, 185	M(2,4,5,6), XM(12,17,19), CY
31	2.32	Kaempferol-3-O-rhamnoside	C_21_H_20_O_10_	-	431.097	285, 284	SJ-Y, M(2,5), XM17, J1, CY, LLM-R
32	8.38	Kaempferol-3-O-glucuronide	C_21_H_18_O_12_	+	463.0858	287	LLM-R
33	8.72	Kaempferol 3-O-arabinoside	C_20_H_18_O_10_	+	419.0969	287, 288, 153	YJ, ZZ
34	12.81	Isorhamnetin-3-O-galactoside	C_22_H_22_O_12_	-	477.105	461, 314	M5, J1, ZZ
	
*Flavanones (9)*							
35	1.79	Farrerol	C_17_H_16_O_5_	-	299.0988	179, 119	SJ-Y, M(1,7), LLM-R
36	7.43	Hesperetin	C_16_H_14_O_6_	+	303.0854	304, 177,153	XM17
37	1.96	Neohesperidin	C_28_H_34_O_15_	-	609.1807	301, 125, 195	SJ-Y, SJ-R, M(1,2,3,4,5,7), XM(1,12,14,17,19), YJ, J1, CY, ZZ, LLM-Y
38	7.62	Naringenin	C_15_H_12_O_5_	-	271.0613	177, 151, 107	SJ-R, M(1,3,4,7), XM(1,17), YJ, CY,
39	2.40	Naringenin-7-O-rutinoside	C_27_H_32_O_14_	-	579.1733	308	SJ-Y, SJ-R, M(1,5), LLM-Y, LLM-R
40	8.75	Kuwanol C	C_25_H_26_O_6_	-	421.1656	367, 45, 123	SJ-Y, M(1,2,3), XM(17,19), ZZ
41	10.18	Liquiritigenin	C_15_H_12_O_4_	-	255.0681	135, 119, 91	SJ-Y, M(1,3,4,6,7), XM14, CY
42	25.14	Liquiritin	C_21_H_22_O_9_	-	417.121	91, 119, 256	SJ-R, M(1,3,4), XM(8,12,14,17,19), YJ, ZZ, LLM-Y, LLM-R


**Compd**	**RT** **(min)**	**Identification**	**Molecular** **formula**	**Model**	**Parents** **ions**	**Fragment ions**	**Sample**
43	10.21	Pinocembrin	C_15_H_12_O_4_	-	255.0653	213, 151	M(4,7)

*Flavones (5)*							
44	2.04	Acacetin-7-O-neohesperidoside	C_28_H_32_O_14_	-	591.1771	575, 471, 427	SJ-Y, SJ-R, M(1,2,3,5,6), XM(1,8,14,17), YJ, J1, CY, ZZ, LLM-R
45	2.17	Gossypetin	C_15_H_10_O_8_	+	319.045	149, 169, 181	M(4,5), XM(1,17), ZZ
46	2.22	Nobiletin	C_21_H_22_O_8_	-	401.1262	373, 388, 355	SJ-Y, SJ-R, M(1,2,3,5,6), XM(1,8,14,17,19), J1, LLM-Y, LLM-R
47	2.99	Tangeretin	C_20_H_20_O_7_	-	371.1159	343, 183	M(2,3,4,5,6,7), XM(8,12,14,17,19), CY, LLM-Y, LLM-R
48	6.43	Bilobetin	C_31_H_20_O_10_	+	553.1124	535, 521, 511	SJ-Y, SJ-R, M(1,2,3,4,5), XM14
49	13.92	Swertisin	C_22_H_22_O_10_	-	445.114	337, 325, 283	SJ-Y, M(1-7), XM(1,8,12,17,19), YJ, J1, ZZ, CY, LLM-Y, LLM-R

*Isoflavone (1)*							
50	2.16	Ononin	C_22_H_22_O_9_	-	475.1266	267	SJ-Y,SJ-R, M(1,3,5,6), XM(1-17), J1, LLM-R

*Ellagitannins (2)*							
51	1.91	Ellagic acid	C_14_H_6_O_8_	-	301.9990	283, 257,	YJ
52	2.21	Chebulagic acid	C_41_H_30_O_27_	-	953.0981	783, 633, 463	XM14, LLM-Y

*Coumarins (3)*							
53	13.98	4-Hydroxycoumarin	C_9_H_6_O_3_	+	163.0394	77, 92, 134	SJ-Y, SJ-R, M(1-7), XM(1-19), YJ, J1, ZZ, CY, LLM-Y, LLM-R
54	1.77	Fraxidin	C_11_H_10_O_5_	+	223.0591	223	SJ-Y, M1
55	6.39	Esculin	C_15_H_16_O_9_	+	341.0854	179, 133, 123	XM17, LLM-R
*Lignan (1)*							


**Compd**	**RT** **(min)**	**Identification**	**Molecular** **formula**	**Model**	**Parents** **ions**	**Fragment ions**	**Sample**
*Lignans (1)*							
56	21.30	Kadsurenin K	C_20_H_22_O_5_	+	365.1367	326, 163, 41	SJ-Y, SJ-R, M(1,2,3,5,6,7), XM(1-19), YJ, J1, ZZ, CY, LLM-Y, LLM-R

*Chalcones (2)*							
57	15.06	Phloretin	C_15_H_14_O_5_	+	275.0899	169, 149, 107	SJ-Y, SJ-R, M(1,3,4,5,6,7), XM(1-19), YJ, J1, ZZ, CY, LLM-Y, LLM-R
58	7.47	Phlorizin	C_21_H_24_O_10_	+	437.1433	419, 275, 169	SJ-Y, M(4,5,6,7), XM(8,12,14,17,19), J1, ZZ, LLM-Y, LLM-R

#### 3.2.1 Hydroxybenzoic acids and derivatives

Compound **1** showed [M+H]^+^ ions at *m/z* 329.0698, as well as fragment ions at 209 [M+H-120 u]^+^, 263 [M+H-66 u]^+^ and 251 [M+H-78 u]^+^, respectively. This compound was identified as bergenin. Compounds **2**, **3**, **4** and **6** at *m/z* 171.0282, 197.8062, 153.0179 and 137.0234, with their resultant ions at *m/z* 125, 153, 109, 137, 79 and 93 respectively, resulting from a loss of CO_2_. The identified compounds were gallic, syringic, protocatechuic and 4-hydroxybenzoic acids, respectively. Compound **5** was determined as 3-O-galloylquinic acid, according to the presence of deprotonated molecules [M-H]^-^ at *m/z* 343.0837 and characteristic ions at *m/z* 191 [M-H-152 u]^-^, indicating losses of galloyl residue (C_7_H_4_O_4_) (Chang et al., 2019).

#### 3.2.2 Hydroxycinnamic acids and derivatives

At *m/z* 337.0899 [C_16_H_16_O_8_+H]^+^, compound **7** produced MS/MS fragment ions at *m/z* 179, 173, 135 and 191. On the other hand, compound **8** presented a predominant peak at *m/z* 337.0811 [C_16_H_18_O_8_-H]^-^. Then, based on their fragmentation pattern, they were characterized as 3-O-caffeoylshikimic acid and 5-*p*-coumaroylquinic acid, consistent with a previous study (Jaiswal et al., 2010; Karar et al., 2014). Compound **9** was recognized as feruloylputrescine, owing to the deprotonated molecules [M-H]^-^ at *m/z* 263.1385, and characteristic ions at *m/z* 57 [M-H-C_11_H_12_NO_3_]^-^, 72 [M-H-C_10_H_9_NO_3_]^-^ and 166 [M-H-C_5_H_9_N_2_]^-^. Compounds **10** (tR 7.09 min, *m/z* 353.0857), **11** (tR 7.76 min, *m/z* 353.0629), **12** (tR 9.11 min, *m/z* 163.0394), **14** (tR 13.52 min, *m/z* 193.0502) and **15** (tR 15.89 min, *m/z* 179.0421) were directly characterized as chlorogenic acid, neochlorogenic acid, *p*-coumaric acid, ferulic acid and caffeic acid by corresponding standards. Compounds **13** had the positively charged molecular ions at *m/z* 375.1057. The fragment ion at *m/z* 179, corresponding to the caffeoyl moiety ([C_9_H_7_O_4_]^+^), arises from cleavage of the central ester bond and is a characteristic marker of caffeic acid esters. The subsequent loss of CO₂ (44 Da) from *m/z* 179 yields the ion at *m/z* 135 ([C₈H₇O₂]^+^), representing the dihydroxy-vinylbenzene cation. Based on these diagnostic fragments, compound **13** was identified as methyl rosmarinate.

#### 3.2.3 Flavan-3-ols

Compound **16** yielded a parent ion at *m/z* 305.066, and the primary fragment ion at *m/z* 125 corresponds to a trihydroxybenzene moiety. It was verified by contrasting it with an earlier report (Liu et al., 2020). Similarly, compound **17** (*m/z* 441.0846) was characterized as (-)-epicatechin gallate. By comparison with the corresponding standards, compounds **18** and **19**, with identical precursors and product ion fragments, were recognized as catechin and epicatechin, respectively. Compounds **20** and **21** were tentatively identified as procyanidins B1 and B2, with the same [M-H]^-^ ions at *m/z* 577. The characteristic ions observed at *m/z* 451 and 425 showed a B arrangement caused by C-C bonding between two (-)-epicatechin molecules (Hong et al., 2019). They also produced ions at *m/z* 289, which is a typical fragment of (-)-epicatechin (Han et al., 2008). Then, these were validated by comparing the retention duration and mass spectrometry results to their standards. Compounds **22** and **23** produced identical excimer ion peaks of [M-H]^-^ at *m/z* 575, two protons less than the B-type procyanidin dimer (Roux et al., 1998). This indicated that they have an extra C-O-C bond in their structure, comparable to the A-type procyanidin dimer. The product ion fragments of compound **22** at *m/z* 539, 407, and 285, respectively. *m/z* 539 was produced by the molecular ion shedding two molecules of H_2_O (36 u). *m/z* 407 was produced by the molecular ion undergoing RDA cleavage (152 u) while shedding one more molecule of H_2_O. *m/z* 285 corresponds to the molecular ion undergoing quinone methide fission (QM) cleavage (290 u). The molecular ion breakage and fragmentation are consistent with the A-type procyanidin dimer cleavage pattern (Hsu et al., 2018; Li et al., 2012). By mass spectrometry database analysis, compounds **22** and **23** were identified as procyanidins A1 and A2, respectively. Similarly, compound **24** was authenticated as procyanidin C1 by comparison with databases and a previous study (Tsugawa et al., 2019).

#### 3.2.4 Flavonols

Compound**25** was observed as positively charged molecular ions at *m/z* 303.0511, and daughter ions at *m/z* 179 and 151, which are characteristic retro Diel-Alder fragments of flavonols. It was identified as quercetin by comparing its fragment ion with the standard. Compound **26** was obtained as a deprotonated molecule [M-H]^-^ with a *m/z* 303.0522 and its product ions at *m/z* 286, 255, 211 and 183, which correspond to the loss of H_2_O, CH_2_O, CO_2_ and CO, respectively. Thus, the compound was identified as (-)-dihydroquercetin, and was verified by comparing the product ion masses reported by Abad-García et al. (2009). Derivatives of quercetin were also found in the jackfruit pulp. Compounds **27** and **28** were determined as 3,7-Di-O-methylquercetin ([M+H]^+^ at *m/z* 353.0633) and quercetin-3-O-glucoside ([M−H]^-^ at *m/z* 463.0885), respectively. The product ion corresponding to the quercetin glycoside element was identified at m/z 301, indicating the loss of the hexoside with a relative molecular mass of 162. Compound **29** at *m/z* 577.1767, was recognized as kaempferitrin according to the characteristic ions at *m/z* 285 [C_15_H_10_O_6_-H]^-^ and 193 [M-C_15_H_10_O_6_-H]^-^ (Li et al., 2022). Compound **30** (kaempferol) exhibited fragment ions at *m/z* 257, 229 and 185, indicating the loss of CH_2_O, 2CHO and C_3_H_2_O_4_. Compound **31** displayed a deprotonated molecule at *m/z* 431.097, along with a rhamnoside moiety [M-H-146 u]^-^ was lost, resulting in product ions at *m/z* 285 [kaempferol-H]^-^ and *m/z* 284 [kaempferol-2H]^-^. It has been identified as kaempferol 3-O-rhamnoside (Elsadig Karar & Kuhnert, 2016). Compounds **32** and **33** were recognized as kaempferol 3-O-glucuronide and kaempferol 3-O-arabinoside, respectively, and the fragment ions at *m/z* 287 [kaempferol+H]^+^ were formed by the loss of glucuronide and arabinoside residues. The precursor ion [M-H]^-^ at *m/z* 477.105 was recognized as isorhamnetin-3-O-galactoside (compound **34**). The loss of hexoside (162 u) units and the aglycone product ion at *m/z* 315 were observed during fragmentation investigations.

#### 3.2.5 Flavanones

Compound **35** was characterized as farrerol, which exhibited fragment ions at *m/z* 179 and 123 as a result of the loss of C_8_H_8_O and C_9_H_8_O_4_, respectively. Considering the precursor ion at *m/z* 303.0854 [C_16_H_14_O_6_+H]^+^, compound **36** was easily identified as hesperitin. Compound **37** showed [M-H]^-^ ion at *m/z* 609.1807, with its fragments observed at *m/z* 301 (aglycone hesperitin), indicating the loss of the glycoside. It was identified as neohesperidin, in accordance with a similar study by Mattonai et al. (2016). Compound **38** was assigned as naringenin due to a deprotonated molecule at *m/z* 271.0613 and fragment ions at *m/z* 177 [M-H-C_6_H_5_OH]^-^, 151 (RDA fragmentation reaction broken at the C-ring of flavonoid aglycones) and 107 [151-CO_2_]^-^ (Wang et al., 2023). Compound **39** showed [M-H]^-^ at *m/z* 579.1733 with a neutral loss of 308 u, compatible with naringenin-7-O-rutinoside. The parent ion [M-H]^-^ at *m/z* 421.1656 and fragments at *m/z* 367 [C_21_H_20_O_6_-H]^-^, 45 [C_2_H_6_O-H]^-^ and 123 [C_7_H_8_O_2_-H]^-^ were used to identify compound **40** as kuwanol C. Both compound **41** and **42** showed fragments at *m/z* 119 [C_8_H_8_O-H]^-^ and 91 [C_8_H_8_O-H-CO]^-^, which have characteristic ions at *m/z* 135, indicating the loss of a phenol group and *m/z* 256, indicating the loss of a hexoside unit, respectively. They were in agreement with liquiritigenin and liquiritin. Additionally, compound **43** exhibited a fragmentation at *m/z* 213 and 151, and was tentatively assigned as pinocembrin.

#### 3.2.6 Flavones

Compound **44** showed a deprotonated molecule at *m/z* 591.1771 and MS/MS spectra with *m/z* 575 [M-H-16 u]^-^, 471 [M-H-120 u]^-^ and 427 [M-H-164 u]^-^. It was found that the disaccharide moiety attached to the aglycone was a neohesperidoside by the ions at *m/z* 471 [M-H-120 u] and 427 [M-H-164 u]. Therefore, this compound may be acacetin-7-O-neohesperidoside after comparing with a report by Lou et al. (2015). Compound **45** with the parent ion at *m/z* of 319.045 [C_15_H_10_O_8_+H]^+^ was determined as gossypetin. Compounds **46** and **47** showed deprotonated molecule ions at *m/z* 401.1262 [C_21_H_22_O_8_-H]^-^ and 371.1159 [C_20_H_20_O_7_-H]^-^, and were assigned as nobiletin and tangeretin, respectively. Compound **48** was characterized as bilobetin based on its fragments at *m/z* 535 [M+H-H_2_O]^+^, 521 [M+H-CH+3OH]^+^, and 511 [M+H-C+2H_2_O]^+^ (Beck & Stengel, 2016). Compound **49** presented [M-H]^-^ ion at *m/z* 445.114 (C_22_H_22_O_10_) with the loss of one C_6_H_10_O_5_ (162 u). Then, it was determined as swertisin.

#### 3.2.7 Isoflavone

One isoflavone (compound **50**) was identified as ononin, based on its molecule ion [M+COOH]^-^ at *m/z* 475.1266 and characteristic ions at *m/z* 267 [M+COOH-162 u]^-^, denoting the loss of glycoside.

#### 3.2.8 Ellagitannins

Ellagitannins, which undergo hydrolysis and liberate a stable ellagic acid, are characterized by the presence of one or more hexahydroxydiphenoyl groups (HHDPs), or their further oxidized forms (Yoshida et al., 2010). Compound **51** showed the precursor ion at *m/z* 301.9990 [M-H]^-^ and the fragments at *m/z* 283 [M-H-H_2_O]^-^, 257 [M-H-CO_2_]- and 229 [M-H-CO_2_-CO]^-^. Hence, it was assigned as ellagic acid. In addition, compound **52** displayed the molecular ion at *m/z* 953.0981 [M-H]^-^, and fragments at *m/z* 783 [M-H-gallic]^-^, 633.0684 [galloyl-HHDP-glucose-H]^-^ and 463.0535 [HHDP-glucose-H-H_2_O]^-^, suggesting that they were likely linked to HHDP glucose and galloyl-HHDP-glucose. Therefore, it was compatible with the reported fragments of chebulagic acid (Li et al., 2021).

#### 3.2.9 Coumarins

Compound **53** exhibited a protonated molecular ion at *m/z* 163.0394. A fragment ion was observed at *m/z* 134, corresponding to the loss of CO (−28 Da) from the coumarin lactone ring. Subsequent cleavage of the heterocyclic core yielded a fragment ion at *m/z* 92, which can be identified as the reverse Diels-Alder ring-opening product C₆H₆O^+^. Additionally, the fragment ion at *m/z* 77 corresponds to the phenyl ion C_6_H_5_^+^, confirming the presence of a benzene ring structure. Therefore, compound **53** is identified as 4-hydroxycoumarin. The identity of compound **54** (C_11_H_10_O_5_) as fraxidin was confirmed by the observation of its protonated molecular ion at *m/z* 223.0591. Compound **55** displayed the molecular ion at *m/z* 341.0854 [M+H]^+^, and MS^2^ fragmentation yielded a prominent ion at m/z 179, corresponding to the aglycone esculetin (loss of C_6_H_10_O_5_, −162 Da), followed by secondary fragments at *m/z* 133 (loss of CO, −28 Da) and *m/z* 123 (ring-opening and H_2_O loss). It was therefore identified as esculin.

#### 3.2.10 Lignans

Compound **56** was identified as kadsurenin K based on the molecular ion at *m/z* 365.1367 [M+Na]^+^. The fragment at *m/z* 326 likely results from the neutral loss of a C_3_H_4_ unit (40 Da), which corresponds to the cleavage of the propenyl group (CH=CH-CH_3_) attached to the aromatic ring. This type of fragmentation is a characteristic feature for lignans bearing a propenyl substituent. *m/z* 163 is a significant and highly diagnostic fragment. It is likely formed through the cleavage of the C7-C8 bond of the lignan backbone. This cleavage would result in a stable fragment ion corresponding to the 3,4-dimethoxyphenyl-propenyl moiety, [C_10_H_11_O_2_]^+^. The ion at *m/z* 41 corresponds to the propenyl cation [C_3_H_5_]^+^, likely formed from the further fragmentation of the molecule or its larger fragments.

#### 3.2.11 Chalcones

Lastly, two chalcones were also identified as phloretin (compound **57**) and phlorizin (compound **58**) by comparing the mass spectrum data with commercial standards. Due to the loss of the phloroglucinol moiety, phloretin exhibited a positively charged precursor ion at *m/z* 275.0899, resulting in *m/z* 169 and 149 [M+H-126 u]^+^. Moreover, phlorizin showed a similar fragmentation pattern to compound **57** and revealed the loss of 18 u (H_2_O) and 162 u (glucose group).

### 3.3 Quantification of phenolic compounds

**Fig. 4:**
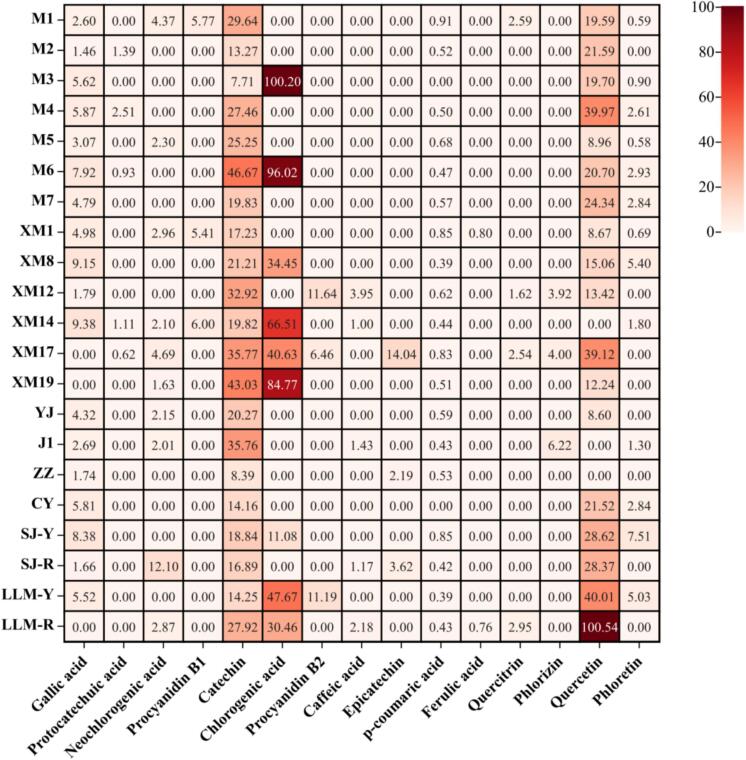
Contents of phenolic compounds in the pulp of different jackfruit cultivars (mg/100 g FW).

To compare the contents of phenolic compounds in the pulp extracted from different cultivars of jackfruit, 15 main phenolics including gallic acid, protocatechuic acid, neochlorogenic acid, procyanidin B1, (+)-catechin, chlorogenic acid, procyanidin B2, caffeic acid, (-)-epicatechin, *p*-coumaric acid, ferulic acid, quercitrin, quercetin, phloretin and phlorizin were quantified (Fig. S2). Meanwhile, specific phenolic compounds were quantified by constructing calibration curves with pure standards of the corresponding phenolic compounds (Table S1). As shown in Fig. 4, different cultivars of jackfruit differed significantly in terms of phenolic content in the pulp. In this study, chlorogenic acid had the highest content among the 15 polyphenols, followed by catechin, quercetin, gallic acid and neochlorogenic acid. The most abundant phenolic acid in jackfruit seeds and peels has also been previously reported to be chlorogenic acid (Fernandes et al., 2017; Jiang et al., 2019). The concentrations of chlorogenic acid in “M3” (100.20 mg/100 g FW), “M6” (96.02 mg/100 g FW) and “XM19” (84.77 mg/100 g FW) were relatively high. In addition, catechin was found in all cultivars, with a mean content of 23.63 mg/100 g FW, and nine cultivars, including M6, XM19, XM17 and J1, had higher catechin levels than the mean. The quercetin content varied significantly, with the highest content of “LLM-R” at 100.54 mg/100g FW, which was significantly higher than the other species. The contents of *p*-coumaric acid were low in different cultivars, with a mean of 0.52 mg/100 g FW.

### 3.4 Antioxidant capacity of extracts

**Fig. 5:**
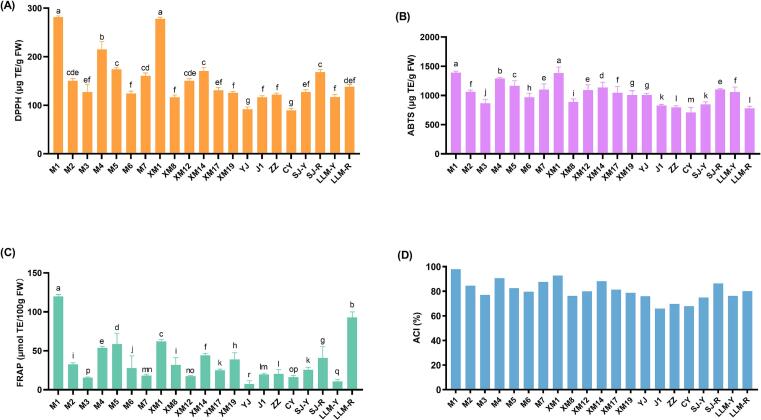
The antioxidant capacity of jackfruit pulp from different cultivars determined by ABTS (A), DPPH (B) and FRAP (C), respectively, as well as the ACI score (D).

The antioxidant capacity among different jackfruit cultivars was investigated using DPPH, ABTS and FRAP assays. As shown in Fig. 5 (A, B and C, different cultivars showed significant differences in antioxidant capacity. Specifically, “XM1” (277.87 g TE/g FW) and “M1” (281.11 g TE/g FW) exhibited the strongest DPPH scavenging capacity, with no noteworthy distinction between the two. Conversely, “CY” (89.21 g TE/g FW) and “YJ” (91.84 g TE/g FW) displayed the lowest scavenging abilities. Similar findings were recorded in the ABTS assay, where “M1” and “XM1” again demonstrated the highest scavenging capacity, with no significant difference observed between them. “CY” showed the weakest ABTS scavenging ability. Additionally, the capacity of “M1” to reduce FRAP (119.75 mol TE/100 g FW) was also significantly better than those of other cultivars, followed by “LLM-R” (92.72 mol TE/100 g FW), while “YJ” (7.14 mol TE/100 g FW) showed the lowest reducing capability.

Since different bioactive compounds may have different affinities in different antioxidant activity assays, and the differing principles underlying each method may yield divergent results, the ACI was used to comprehensively assess the antioxidant capacity of each extract (Lin et al., 2022). As shown in Fig. 5D, M1 achieved the highest ACI, followed by “XM1”, “M4”, “XM14” and “M5”, among others.

### 3.5 Relationship between the phenolics content and antioxidant capacity

**Fig. 6:**
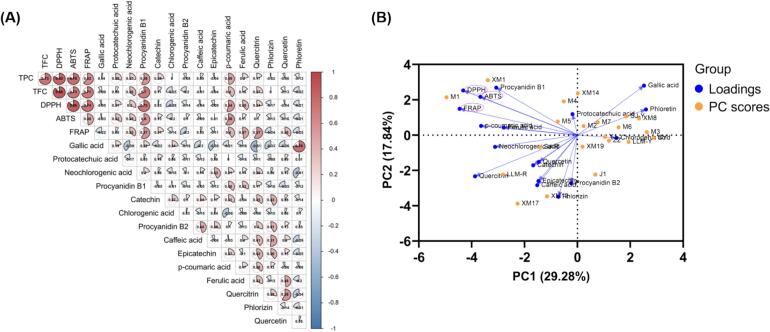
Correlation analysis matrix (A) and PCA biplot (B) of phenols content and antioxidant capacity. (A) An increasing degree of redness indicates an increasing positive correlation and an increasing degree of blueness indicates an increasing negative correlation. (B) The percentage of circles indicates the magnitude of the correlation. The orange dot represents each sample of jackfruit.

Previous studies have shown a correlation between the antioxidant activity of fruits and vegetables and their phenolic content (Amagloh et al., 2022; Zhang, Zhu, et al., 2021). As seen in Fig. 6A, the antioxidant capabilities measured by ABTS, DPPH, and FRAP assays were all significantly and positively linked with TPC and TFC (*p* < 0.05). Specifically, approximately 78% and 73% of the variation observed in ABTS antioxidant capacity was associated with TPC and TFC, respectively, compared to about 82% and 88% in DPPH, and about 62% and 71% in FRAP. These results suggest that TPC and TFC exert a more pronounced effect on DPPH antioxidant activity compared to the other assays. In addition, procyanidin B1 and *p*-coumaric acid accounted for more than half of the variation in antioxidant capacity, suggesting these metabolites may be the primary contributors to the antioxidant capacity of jackfruit pulp. Procyanidins are the most powerful free radical scavenging natural products, and the large number of hydroxyl groups in their structure is the material basis for their antioxidant and free radical scavenging properties. *P*-coumaric acid has a central role in the secondary metabolism of plants and is widely found in plant foods, such as fruits, vegetables, tea, coffee and cereals (Pei et al., 2016), and the antioxidant effect is also an important basis for other pharmacological effects of *p*-coumaric acid.

A comprehensive evaluation of 21 jackfruit cultivars was performed based on principal component analysis (PCA). The results were shown in a biplot (Fig. 6B) that integrated various independent variables and different samples. The first and second principal components (PC) accounted for 47.12% of the total variance in the dataset. The differences in the dominance of various jackfruit samples for various phenolic compounds were evident. The cosine of the angle between any two vectors estimates their pairwise correlation (acute angles for positive correlation, right angles for no correlation, obtuse for negative correlation), while the length of each vector is proportional to the variable’s loading on the first two principal components, representing its relative contribution to the explained variance. The proximity of variables indicates that they differ little in quantity or level and that similarity exists. Additionally, the distinct variations in the dominance of various phenolic compounds across samples are clearly evident. For example, “M1” and “XM1” exhibited similar distributions, however, they significantly differed from other varieties due to their elevated levels of proanthocyanidin B1, coumaric acid, TPC, TFC, and antioxidant capacity.

## 4 Discussion

Jackfruit is well recognized for its rich nutrient composition, including carbohydrates, proteins, vitamins, and its abundance of bioactive phenolics, which confer antioxidant, anti-inflammatory, and other health benefits (Dhani et al., 2025). Jackfruit fruit and peel polyphenols have been shown to mitigate oxidative stress and inflammatory markers in vitro, supporting their use in functional foods and nutraceuticals (Gupta et al., 2023). The selection of superior cultivars is therefore a critical strategy for maximizing these benefits for human health. This study represents the first systematic evaluation of TPC, TFC, individual polyphenolic profiles, and antioxidant capacity across 21 jackfruit cultivars, highlighting the critical role of cultivar selection in determining nutritional value and health-promoting potential of this tropical fruit.

As key quality metrics, TPC and TFC reflect the abundance of phenolic compounds and correlate strongly with antioxidant capacity, flavor attributes, and health-promoting effects in fruits. Our findings showed a remarkable difference in TPC and a similar variance in TFC across cultivars, with “M1” and “XM1” consistently ranking among the highest. These results significantly surpass the values reported in earlier studies on single cultivars (Jagtap et al., 2010). Differences in content between varieties may stem from the inherent genetic background of the cultivars. Distinct genetic blueprints dictate the expression levels and activity of key enzymes within the phenylpropanoid biosynthetic pathway, such as phenylalanine ammonia-lyase (PAL) and chalcone synthase (CHS), thereby governing the differential accumulation and composition of phenolic compounds (Zagoskina et al., 2023).A core discovery of this research was the profound qualitative and quantitative variation in phenolic profiles across the germplasm. Our untargeted metabolomic approach identified 58 distinct phenolic compounds, far exceeding previous reports and allowing for a nuanced understanding of their distribution. The phenolic fingerprint defines the functional signature of each jackfruit cultivar. Among the 58 identified phenolic compounds, hydroxybenzoic acids (e.g., gallic acid), hydroxycinnamic acids (e.g., chlorogenic acid), and flavonoids (e.g., quercetin) were universally detected, suggesting they play fundamental physiological roles and constitute a basal chemical signature for the species. In stark contrast, we also discovered cultivar-specific compounds. For instance, 4-Hydroxybenzoic acid (in “XM12”), Procyanidin A1 (in ‘M4’), and Ellagic acid (in “YJ”) function as potential chemotaxonomic markers. Furthermore, our analysis revealed the presence of various phenolic compounds in cultivars including “M1”, “XM1”, and “LLM-R”. These cultivars are of particular interest for developing functional foods with potentially synergistic health benefits derived from a complex mixture of phenolics.

The strong positive correlations observed between TPC/TFC and antioxidant activity are consistent with established literature. However, the varying strength of these correlations across different assays warrants a deeper mechanistic interpretation. The DPPH assay operates predominantly *via* a Hydrogen Atom Transfer (HAT) mechanism, which directly measures the capacity of a compound to donate a hydrogen atom (Przybylski et al., 2022). It is highly sensitive to phenolics with ortho-dihydroxy groups (e.g., flavan-3-ols like procyanidin B1 and flavonols like quercetin). This structural specificity aligns with jackfruit’s dominant phenolic classes, explaining the robust correlation. In contrast, the ABTS assay functions through a mixed mode of both HAT and Single Electron Transfer (SET), allowing it to react with a broader spectrum of antioxidants, which may dilute its specific correlation with phenolic content alone (Floegel et al., 2011). The FRAP assay, being a pure SET-based method, exclusively measures the reducing power of a sample (i.e., its ability to donate an electron) and not its hydrogen-donating capacity (Firuzi et al., 2005). When plant extracts are rich in phenolics and flavonoids, the DPPH assay shows particularly high sensitivity. For instance, in cucurbit fruits extracts, DPPH showed much stronger correlations with TPC and TFC (r = 0.959 and 0.936, respectively) compared to ABTS (r = 0.948) (Singh et al., 2016).

Notably, Procyanidin B1 and *p*-Coumaric acid emerged as primary contributors to antioxidant capacity. The role of procyanidin B1 is likely due to its dimeric structure, which provides more radical-scavenging hydroxyl groups than its monomeric counterpart, (+)-catechin. Similarly, the influence of p-coumaric acid, despite its low concentration, underscores that high chemical reactivity, not just quantity, dictates bioactivity. Studies have shown that the unsaturated side chain of *p*-coumaric acid may enhance membrane permeability, facilitating interaction with radical species (Zanardo et al., 2009). PCA further clustered the cultivars according to their phenolic composition and antioxidant characteristics, grouping “M1”and “XM1” together on account of their enriched bioactive profiles and elevated antioxidant indices. This finding suggests that these two cultivars represent ideal parental genotypes for functional food breeding programs aimed at enhancing antioxidant capacity.

Jackfruit cultivars differ markedly in their polyphenol composition and antioxidant assay responses. Key compounds such as procyanidin B1 and p-coumaric acid contribute unevenly to overall bioactivity. These interrelated trait correlations vary by cultivar. Understanding them is essential for guiding breeding and selection strategies aimed at improving both nutritional value and functional quality.

## 5 Conclusion

This study compared the phenolic profiles and antioxidant activities of jackfruit pulp from 21 different cultivars. Utilizing UPLC-Q-TOF-MS/MS, a total of 58 phenolic compounds were identified, confirming that jackfruit pulp is a rich source of phenolics. Cultivars with higher total phenolic content (TPC) exhibited stronger antioxidant activity. Procyanidins B1 and *p*-coumaric acid emerged as key metabolites influencing the antioxidant activity. Notably, the “M1” cultivar demonstrated the highest TPC, TFC, and antioxidant capacity. These findings provide valuable insights into the bioactive benefits of different jackfruit cultivars. However, this study is limited to analysis of fruit pulp only. Peel and seed tissues may exhibit different phenolic profiles and activities. To fully realize the potential of jackfruit as a functional food, Future work should characterize multi-tissue metabolomes to assess whole-fruit antioxidant potential, and further investigation into its biological activities and the underlying mechanisms need to be further studied.

## CRediT authorship contribution statement

**Ming Cheng:** Writing – review & editing, Writing – original draft, Methodology, Investigation, Formal analysis, Data curation. **Mengyang Liu:** Methodology, Investigation, Data curation. **Lehe Tan:** Data curation. **Chuan Li:** Resources. **Gang Wu:** Methodology. **Bingqiang Xu:** Validation, Resources, Funding acquisition. **Yanjun Zhang:** Methodology, Investigation. **Kexue Zhu:** Supervision, Project administration, Funding acquisition.

## Funding

This work was supported by the Key Research and Development Project of Hainan Province (No. ZDYF2024HXGG002, No. ZDYF2024XDNY277), the Central Public-interest Scientific Institution Basal Research Fund for Chinese Academy of Tropical Agricultural Sciences (No. 1630012025118, No. 1630142022009), the Project of State Key Laboratory of Tropical Crop Breeding (No. NKLTCB202342), and the Chinese Academy of Tropical Agricultural Sciences for Science and Technology Innovation Team of National Tropical Agricultural Science Center (NO. CATASCXTD202304).

## Declaration of competing interest

The authors declare that they have no known competing financial interests or personal relationships that could have appeared to influence the work reported in this paper.

## Data Availability

Data will be made available on request.

## Appendix A Supplementary data

Supplementary material Image 1
