# Supplementary material for: Comparison of phenolic profiles and antioxidant activities in the pulps from 21 different Artocarpus heterophyllus Lam. cultivars
Source: Food Chem X. 2025 Jul 4;29:102735. doi: 10.1016/j.fochx.2025.102735 (PMC12274677; doi:10.1016/j.fochx.2025.102735)
Supplement: Supplementary file 1 — Supplementary material [file mmc1.docx]

**Comparison of phenolic profiles and antioxidant activity of** **pulp from** **21 different *Artocarpus heterophyllus* Lam. cultivars**

Ming Cheng ^a, b, c^, Mengyang Liu ^a, e, f^, Lehe Tan ^a^, Chuan Li ^b^, Gang Wu ^a, e^, Bingqiang Xu ^a, d *^,Yanjun Zhang ^a, e, f^, Kexue Zhu ^a, e, f *^

^a^ National Key Laboratory for Tropical Crop Breeding, Spice and Beverage Research Institute, Chinese Academy of Tropical Agricultural Sciences，Sanya/Wanning Hainan 572024/571533，China

^b^ School of Food Science and Engineering, Hainan University, Haikou, Hainan 570228, China

^c^ School of Food Science and Engineering, Ocean University of China, Qingdao, Shandong 266003, China

^d^ Sanya Research Institute of Chinese Academy of Tropical Agricultural Sciences, Chinese Academy of Tropical Agricultural Sciences, Sanya Hainan 572024, China

^e^ Key Laboratory of Processing Suitability and Quality Control of the Special Tropical Crops of Hainan Province, Wanning, Hainan 571533, China

^f^ National Center of Important Tropical Crops Engineering and Technology Research, Wanning, Hainan 571533, China

* Corresponding author:

erger2002@163.com (Prof. Bingqiang Xu),

zhukexue163@163.com (Prof. Kexue Zhu).

| **Compound** | **Linear range (mg/L)** | **Regression equation** | **R^2^** | **LOD (mg/kg)** |
| --- | --- | --- | --- | --- |
| Gallic acid | 25-200 | y = 16.592x+5.117 | 0.999 | 0.12 |
| Protocatechuic acid | 25-200 | y = 26.597x-104.15 | 0.999 | 0.12 |
| Neochlorogenic acid | 25-200 | y = 1.2793x-23.599 | 0.997 | 0.12 |
| Procyanidin B1 | 12.5-200 | y = 0.0206x-0.1386 | 0.999 | 0.50 |
| Catechin | 25-200 | y = 0.7771x-10.353 | 0.999 | 0.25 |
| Chlorogenic acid | 25-200 | y = 5.5303x-104.93 | 0.997 | 0.12 |
| Procyanidin B2 | 25-200 | y = 0.0753x-1.0439 | 0.999 | 0.50 |
| Caffeic acid | 12.5-200 | y = 42.792x-412.74 | 0.999 | 0.12 |
| Epicatechin | 25-200 | y = 8.5256x-56.872 | 0.993 | 0.50 |
| *p*-Coumaric acid | 12.5-200 | y = 71.397x-562.35 | 0.999 | 0.12 |
| Ferulic acid | 12.5-200 | y = 41.294x+99.387 | 0.993 | 0.12 |
| Quercitrin | 12.5-200 | y = 10.789x-61.646 | 0.999 | 0.12 |
| Phlorizin | 12.5-200 | y = 12.181x+66.713 | 0.999 | 0.12 |
| Quercetin | 12.5-200 | y = 16.686x-98.051 | 0.999 | 0.25 |
| Phloretin | 12.5-200 | y = 44.216x-354.12 | 0.999 | 0.12 |

**Table S1.** Linear range, regression equation, R^2^ and LOD of fifteen compounds.

**Fig. S1.** MS spectra and chemical structures of the 58 phenolic compounds identified.


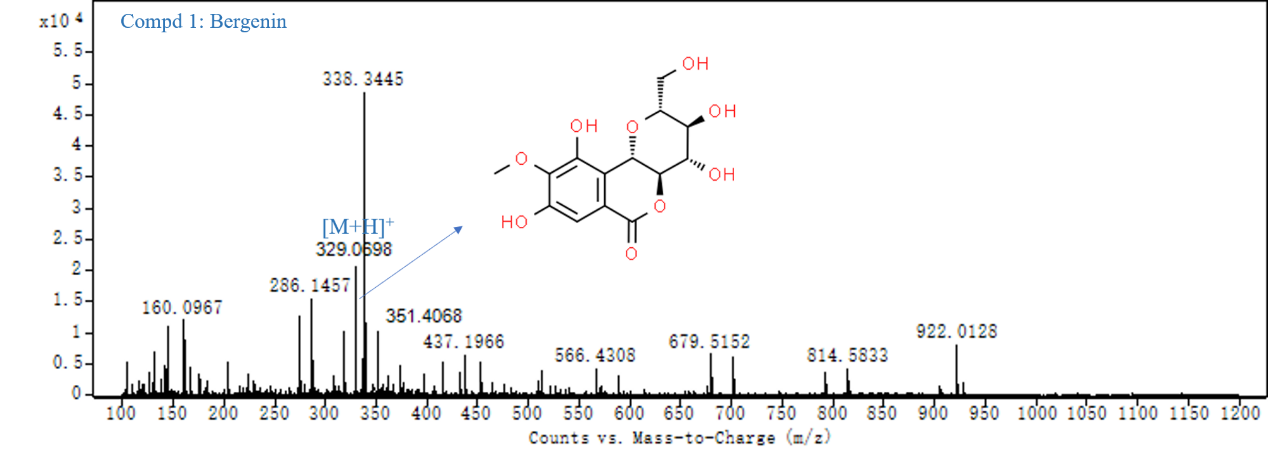


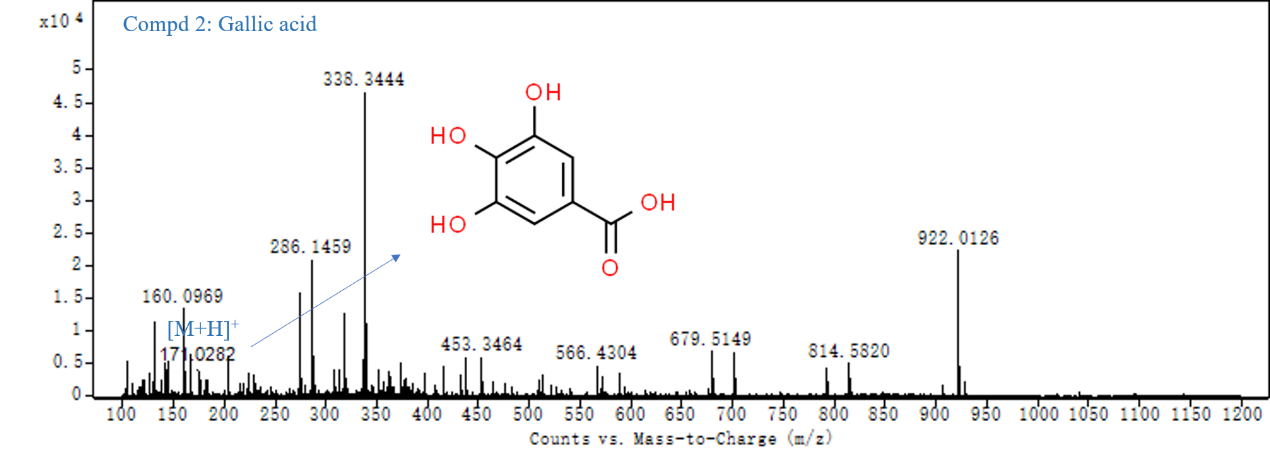


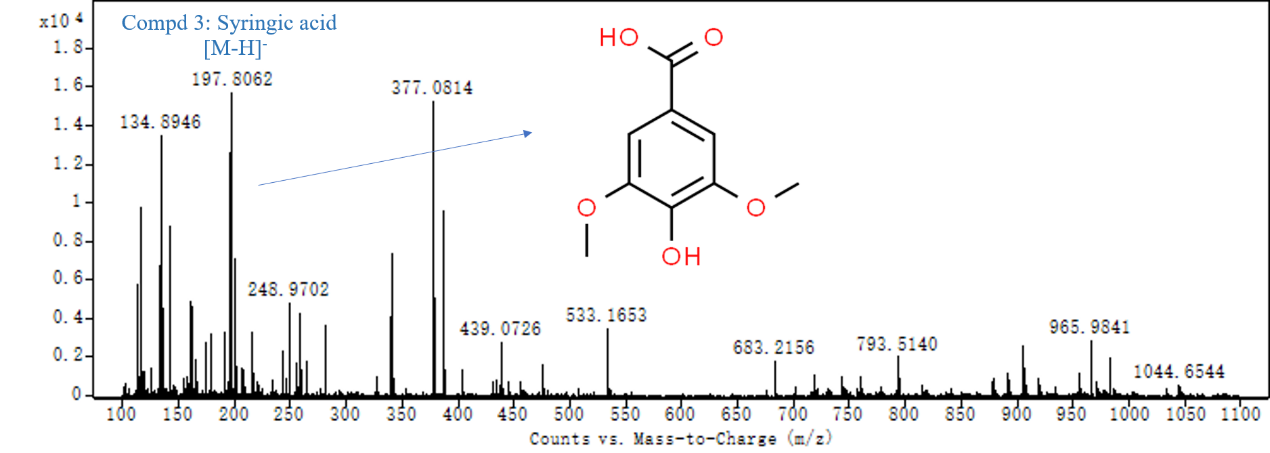


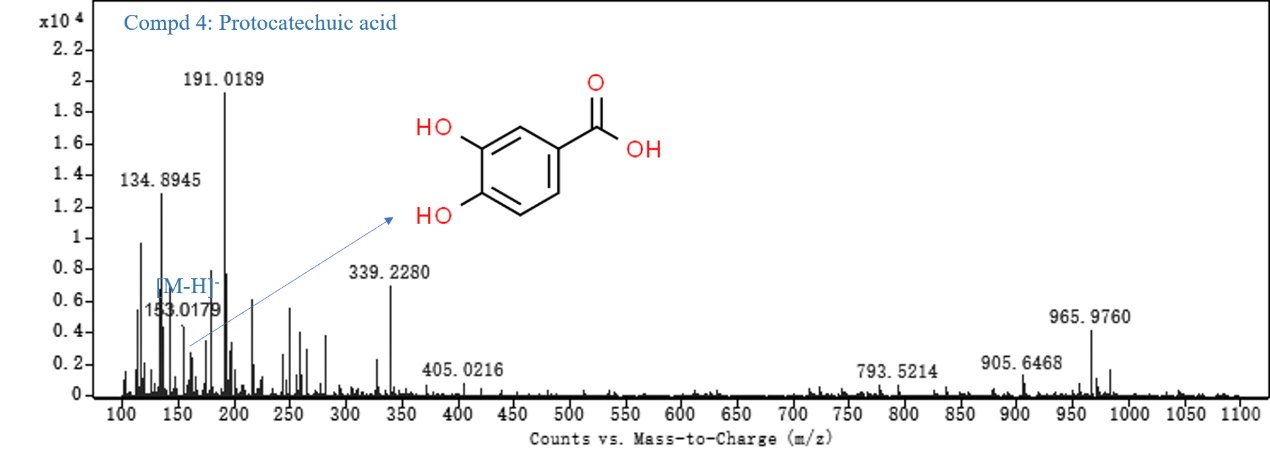


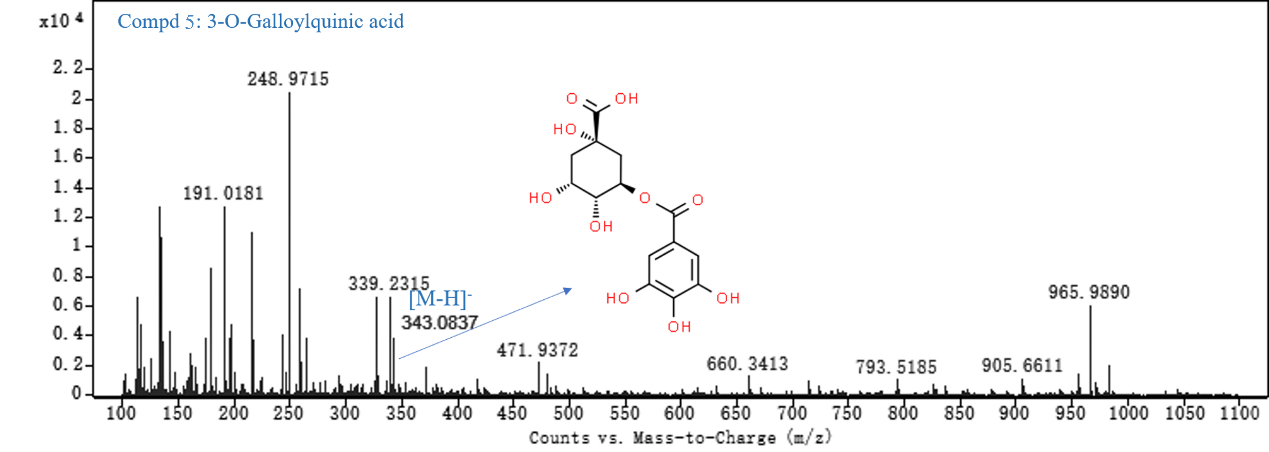


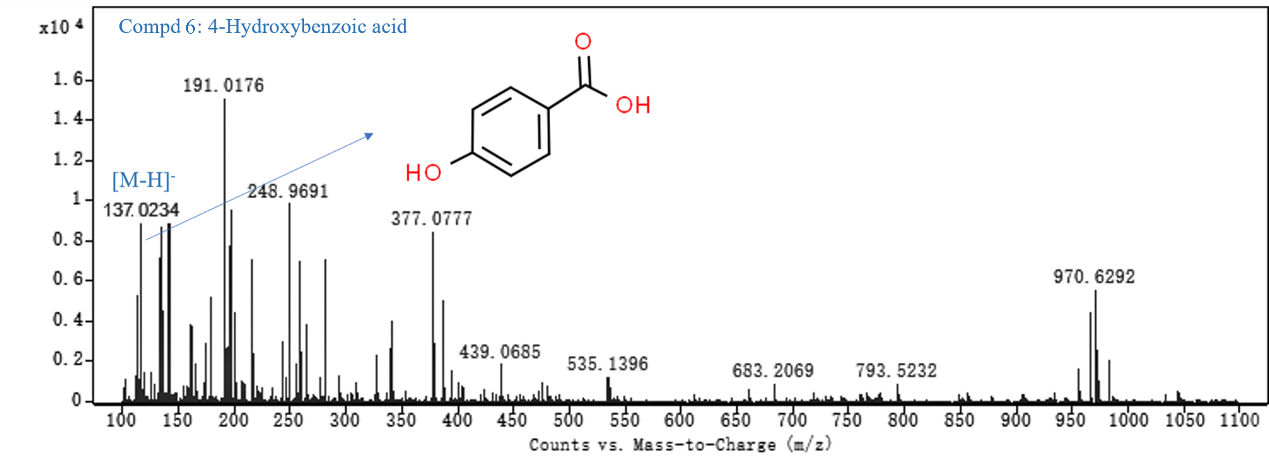


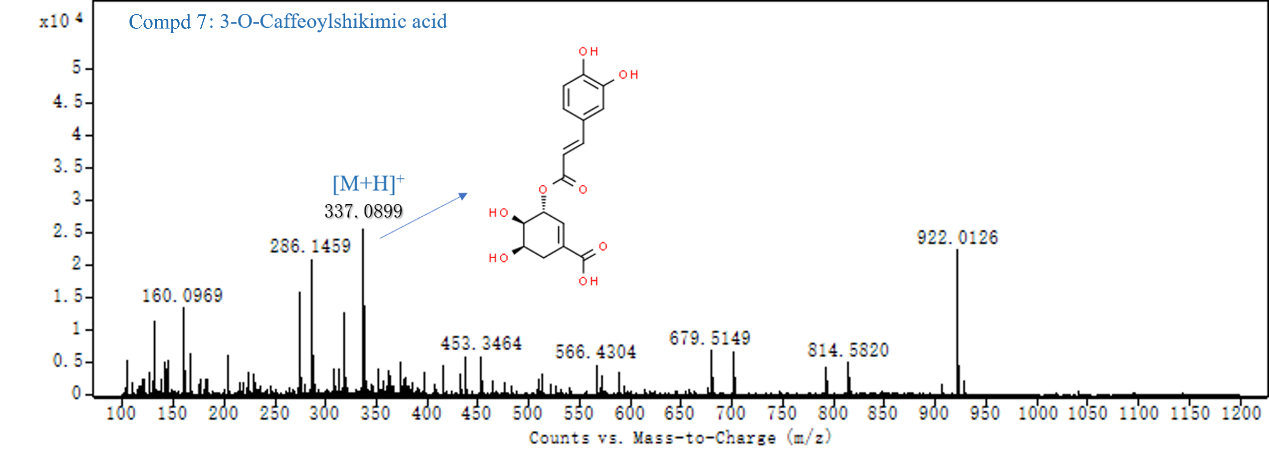


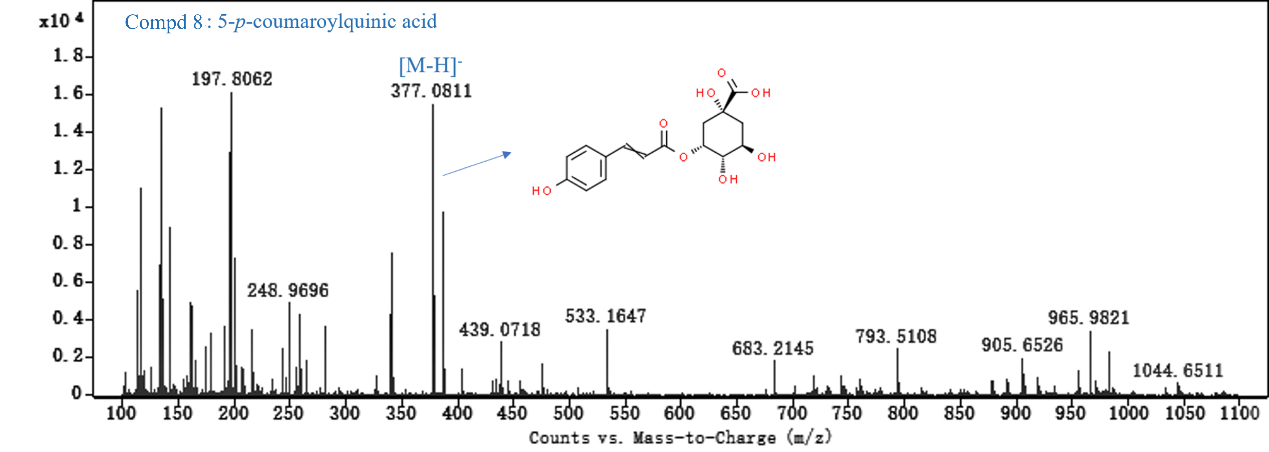


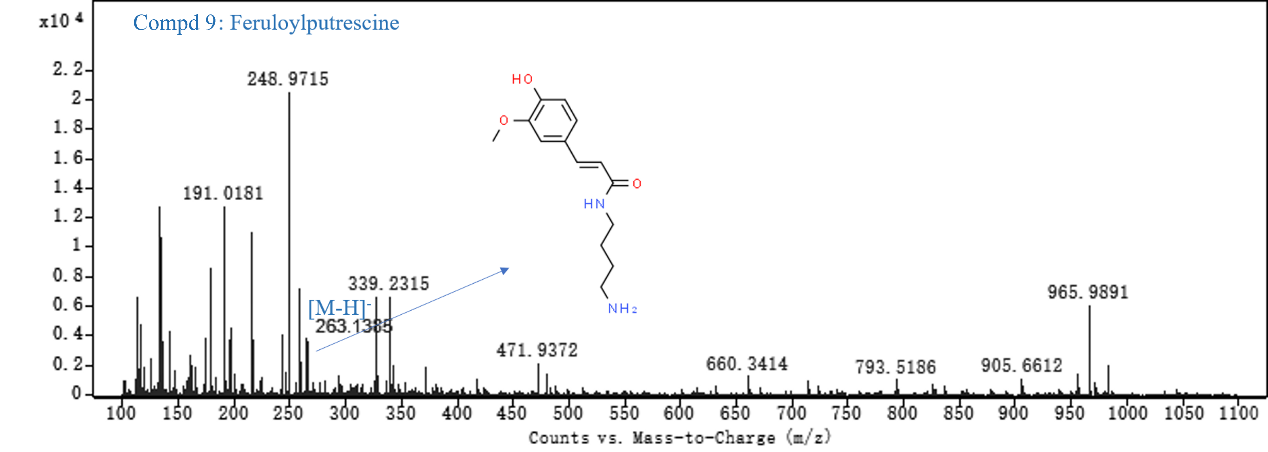


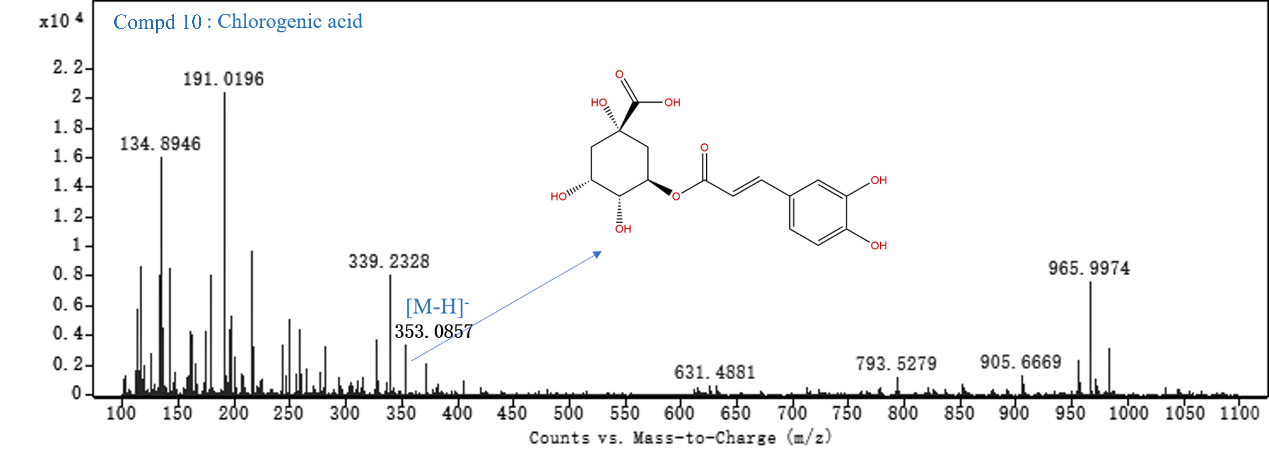


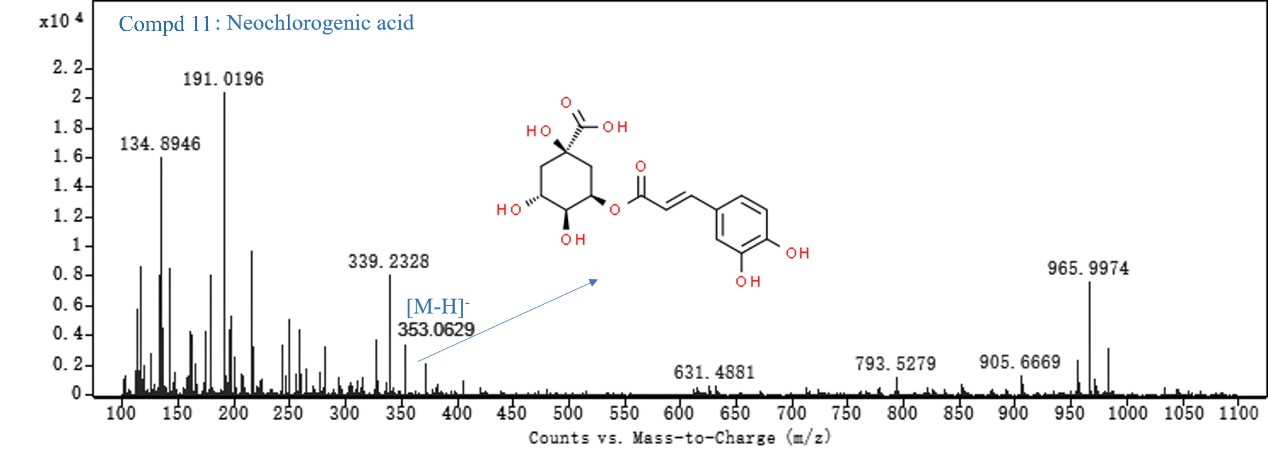


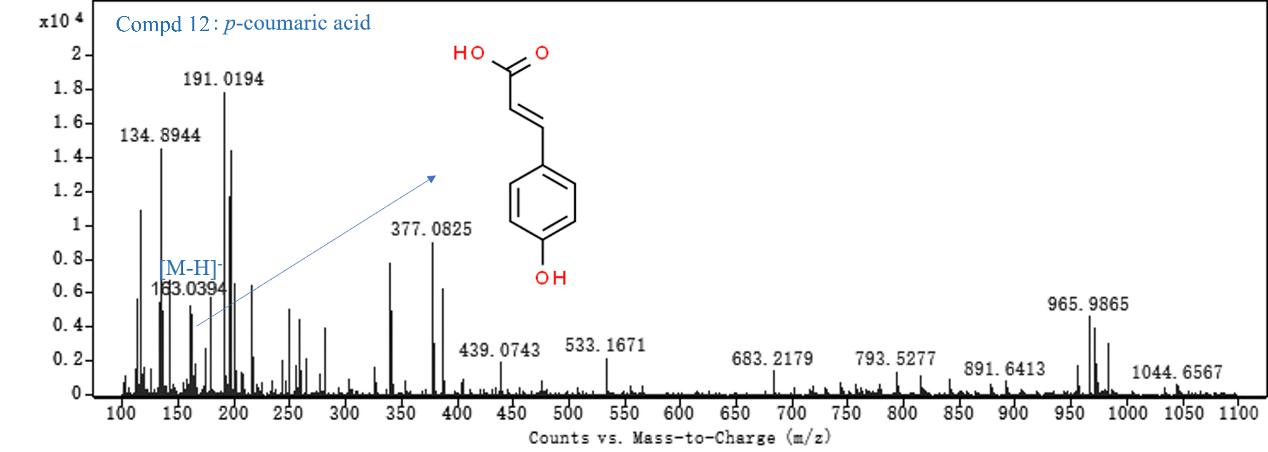


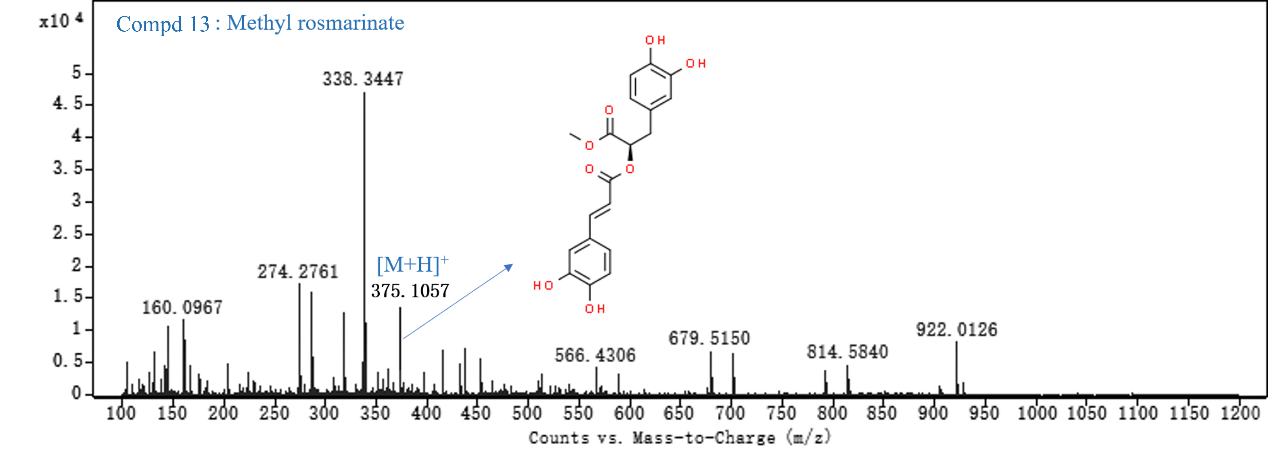


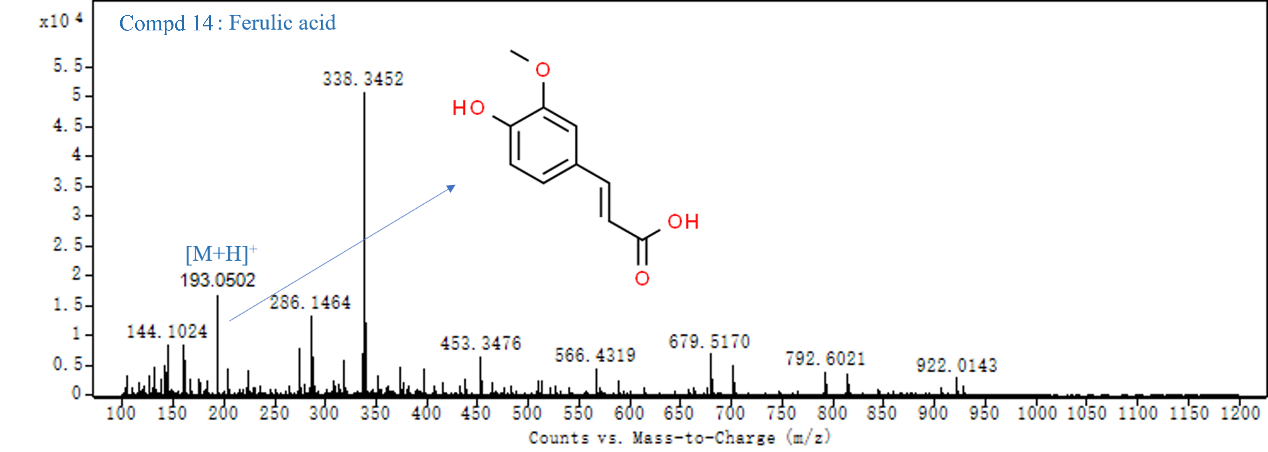


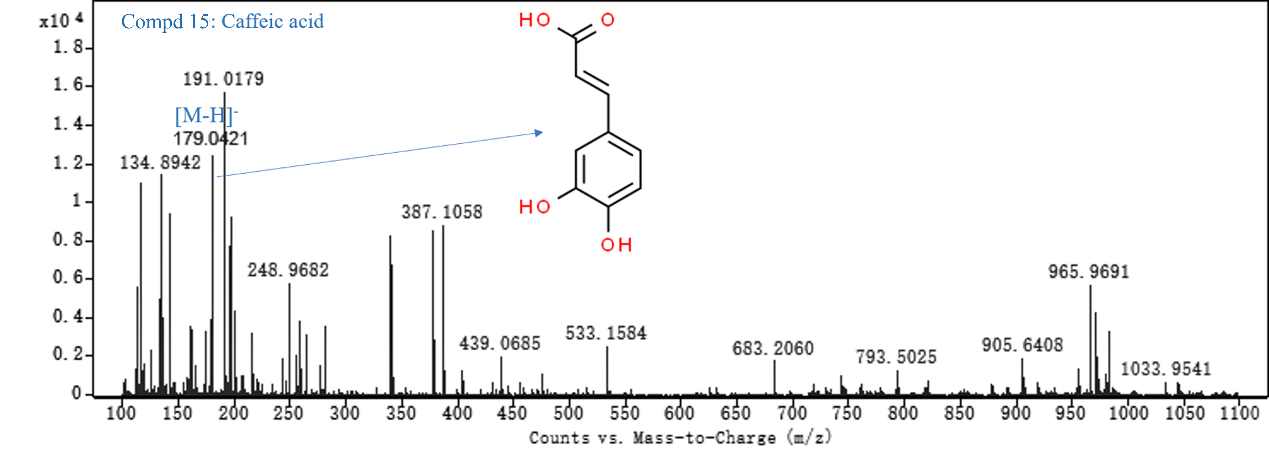


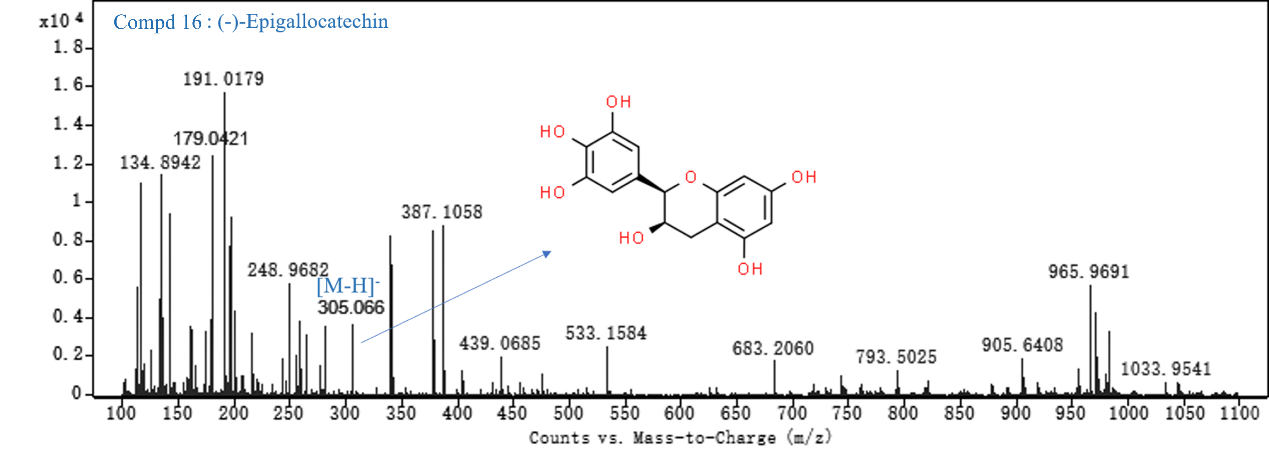


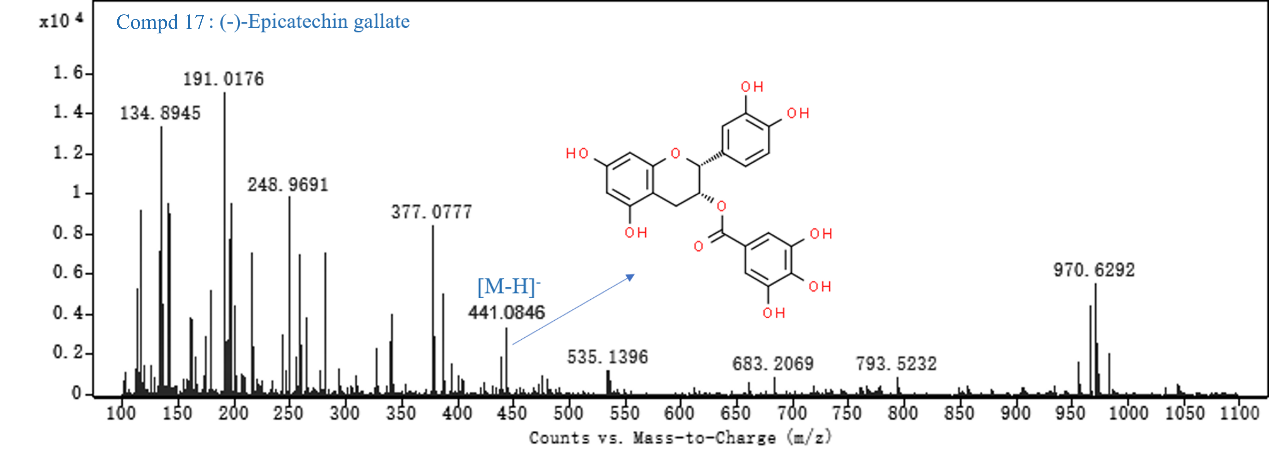


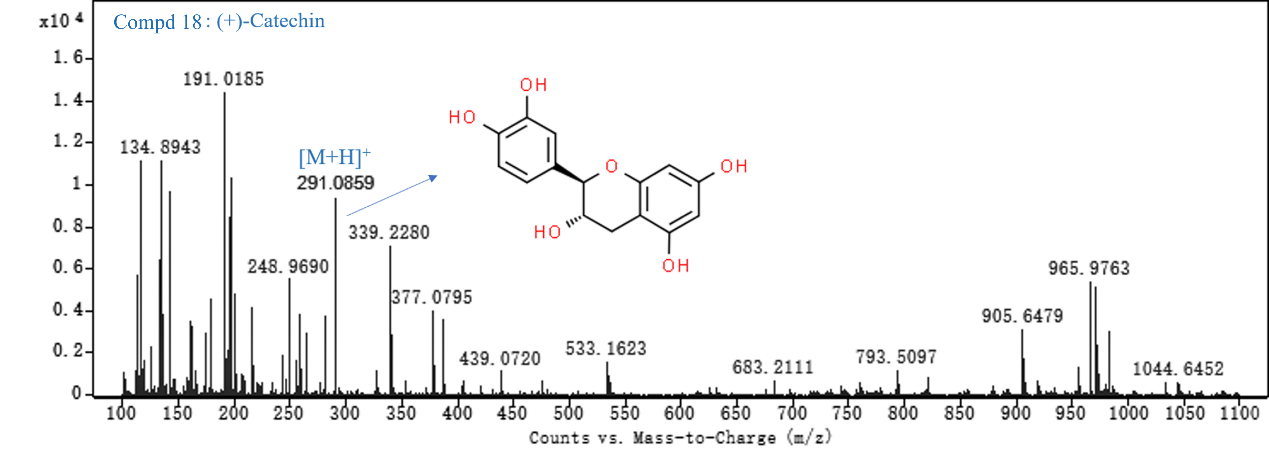


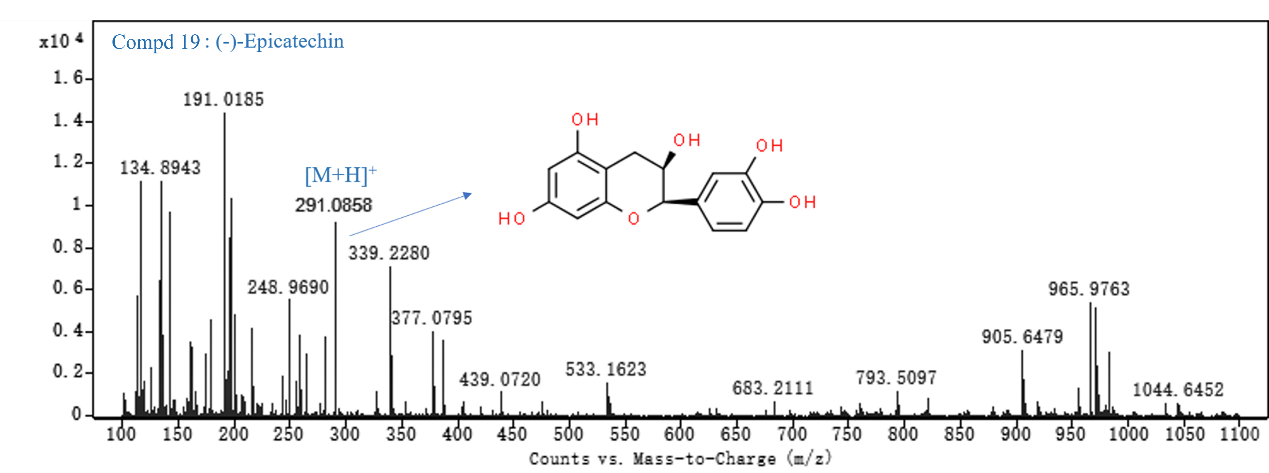


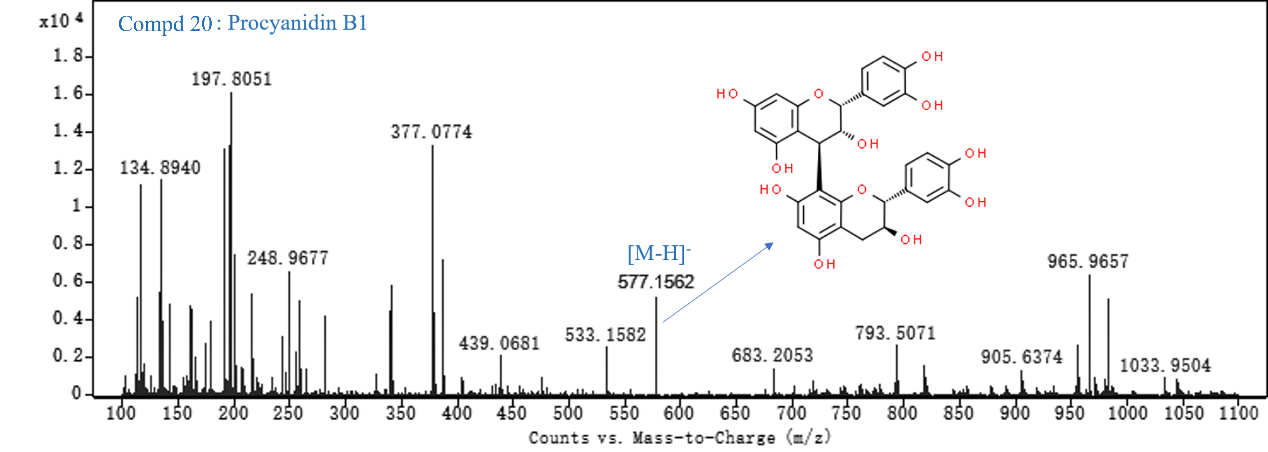


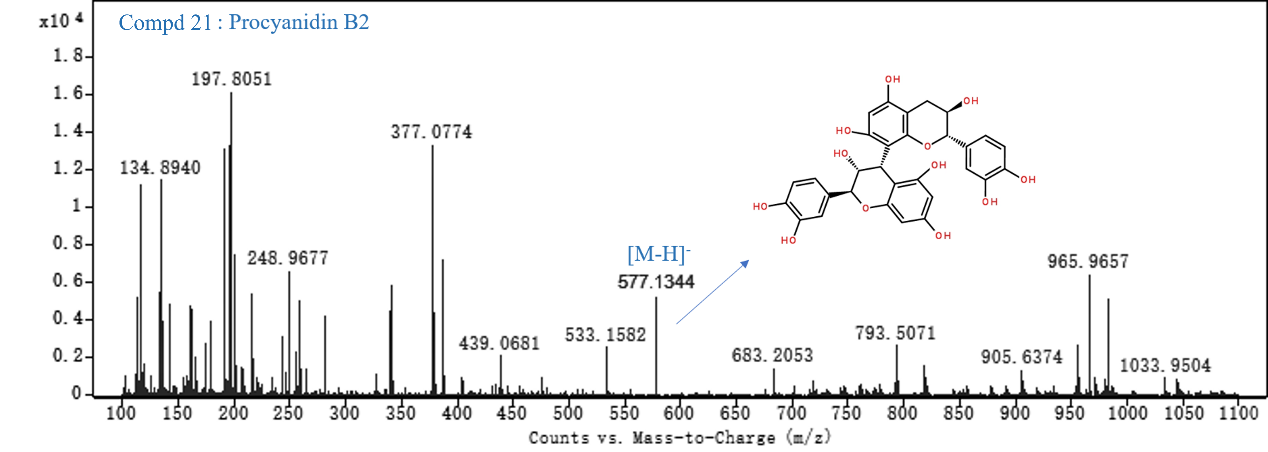


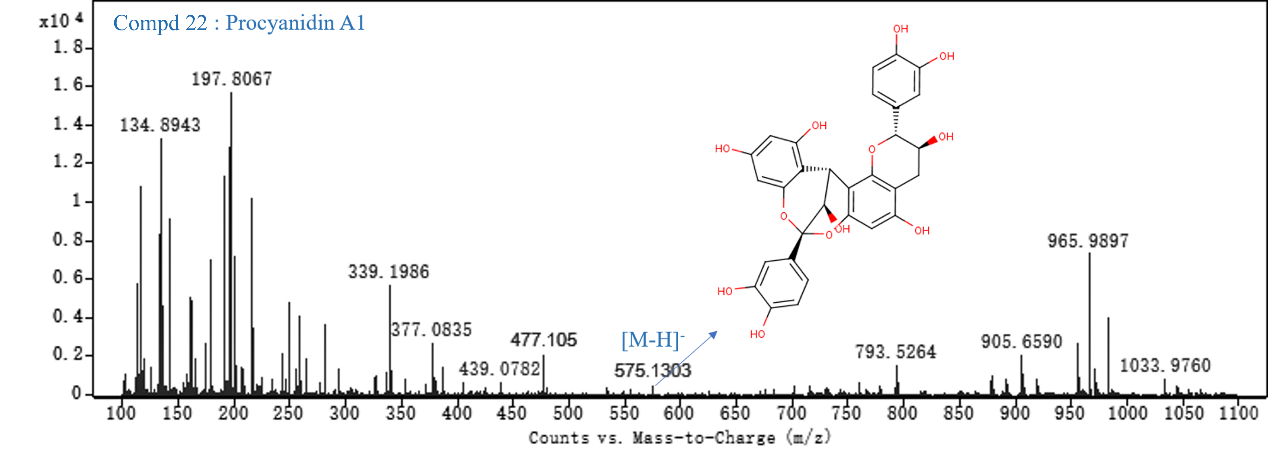


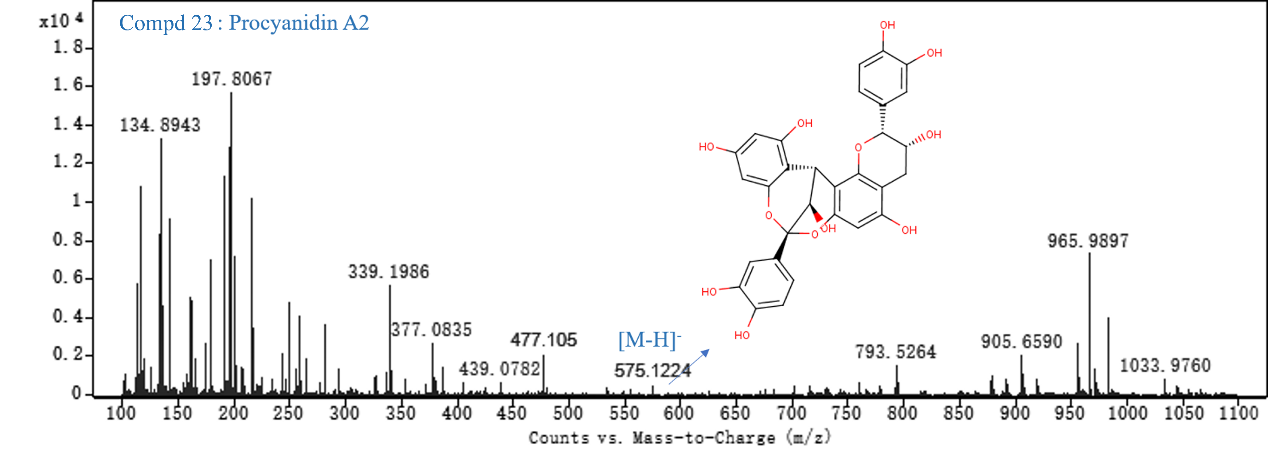


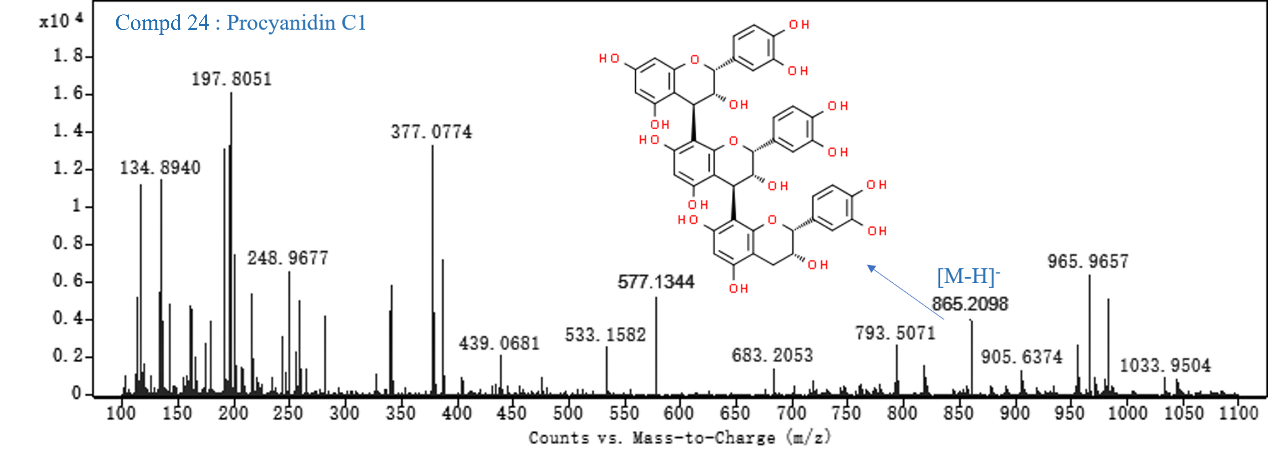


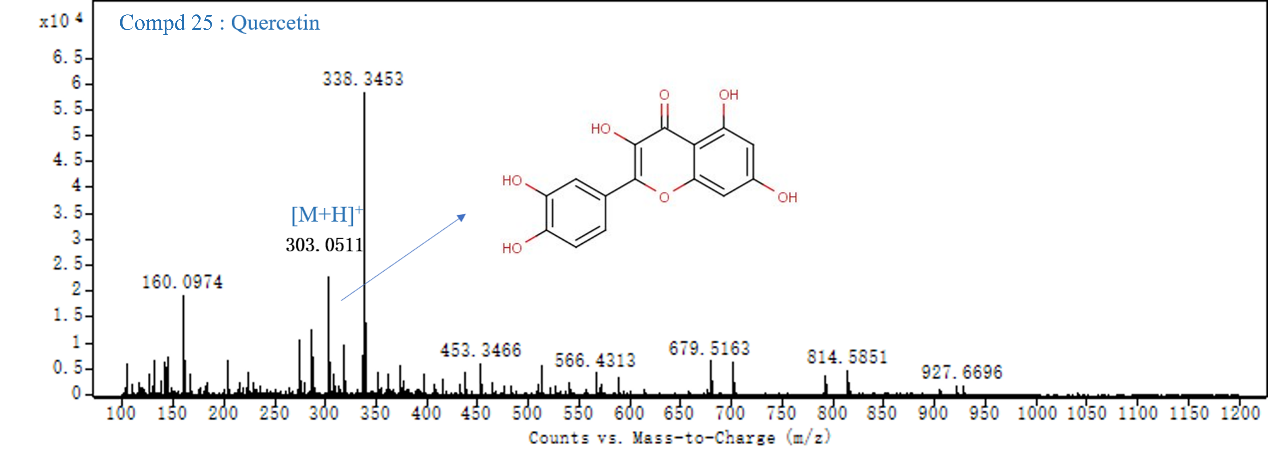


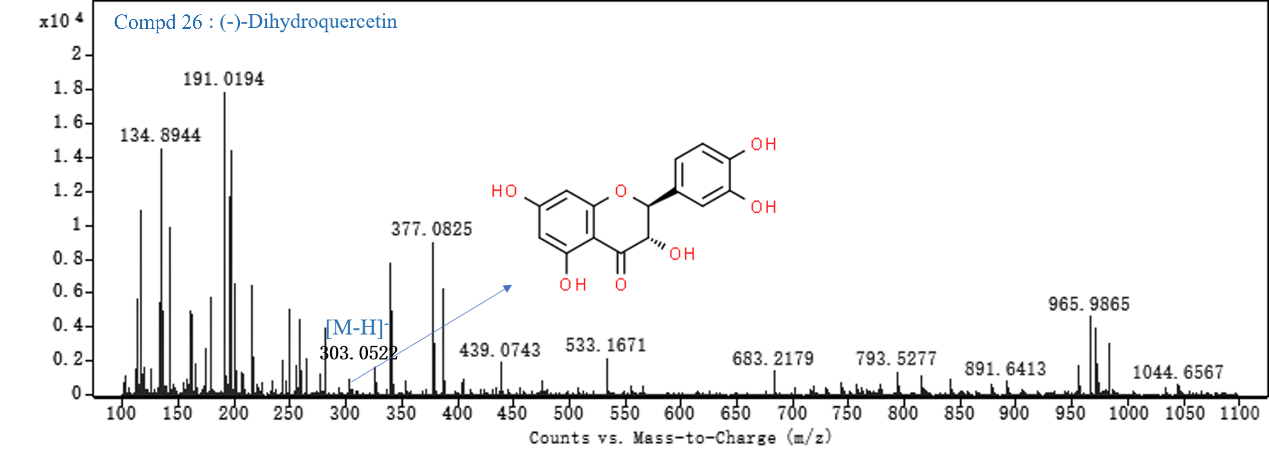


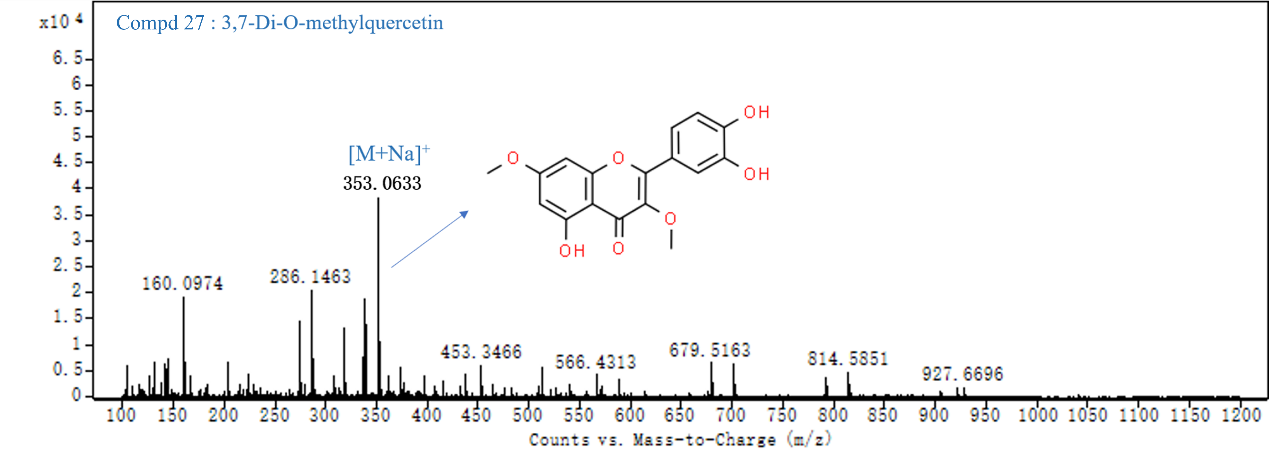


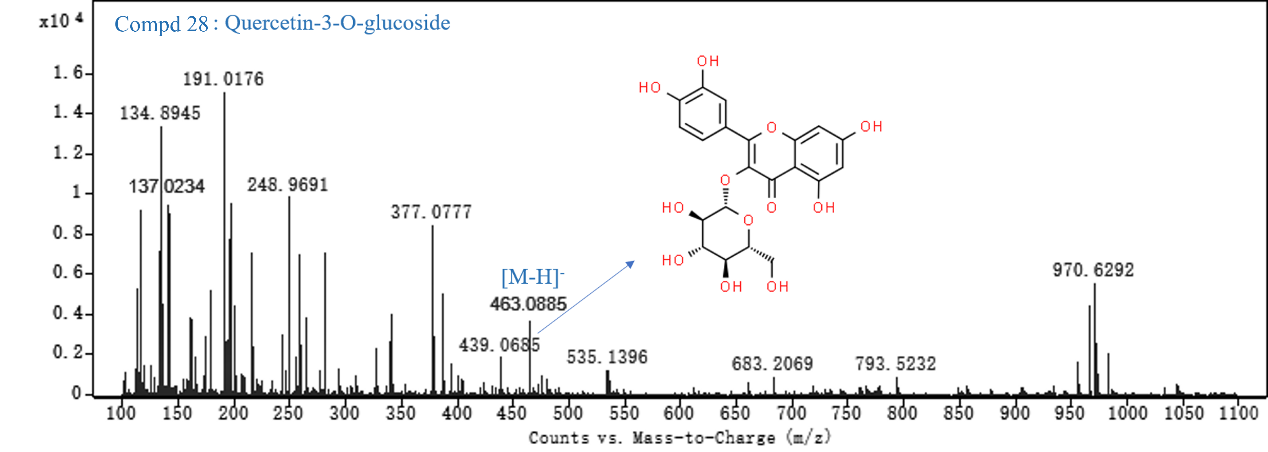


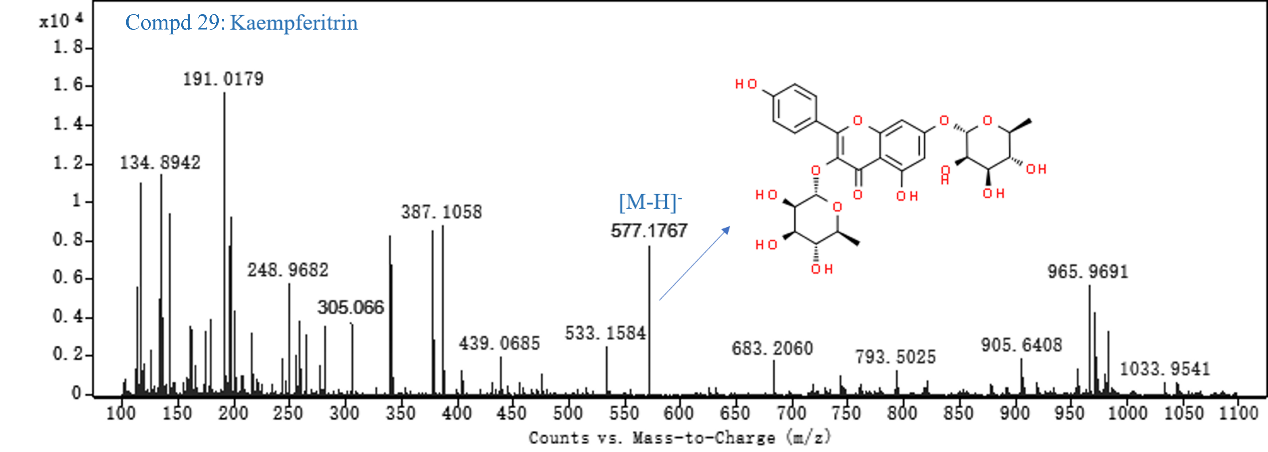


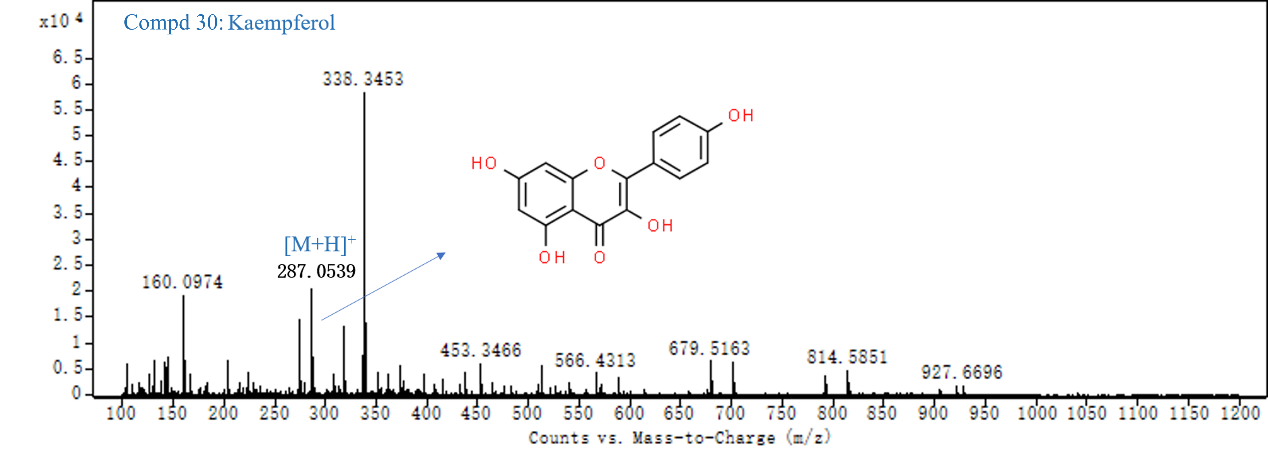


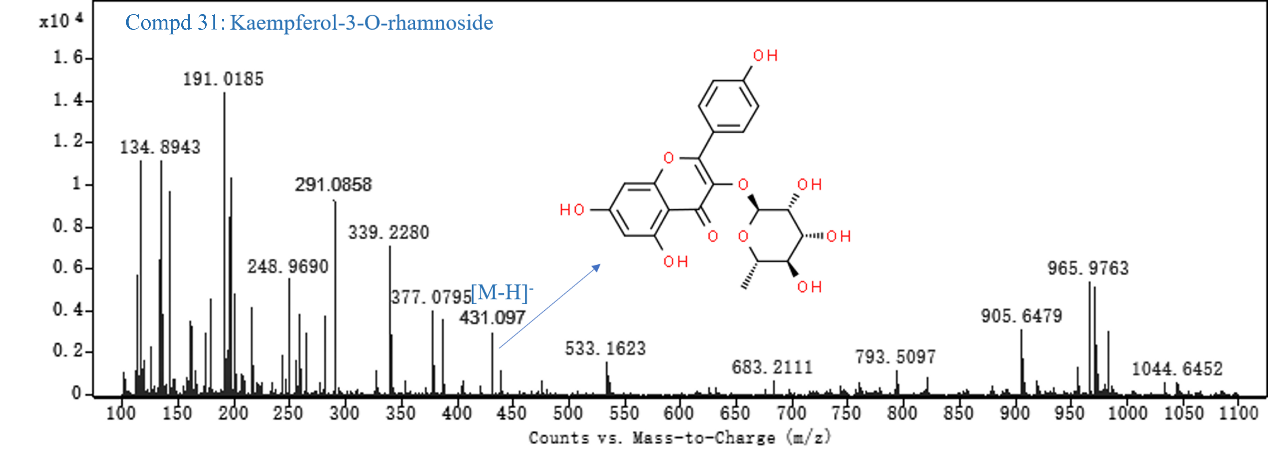


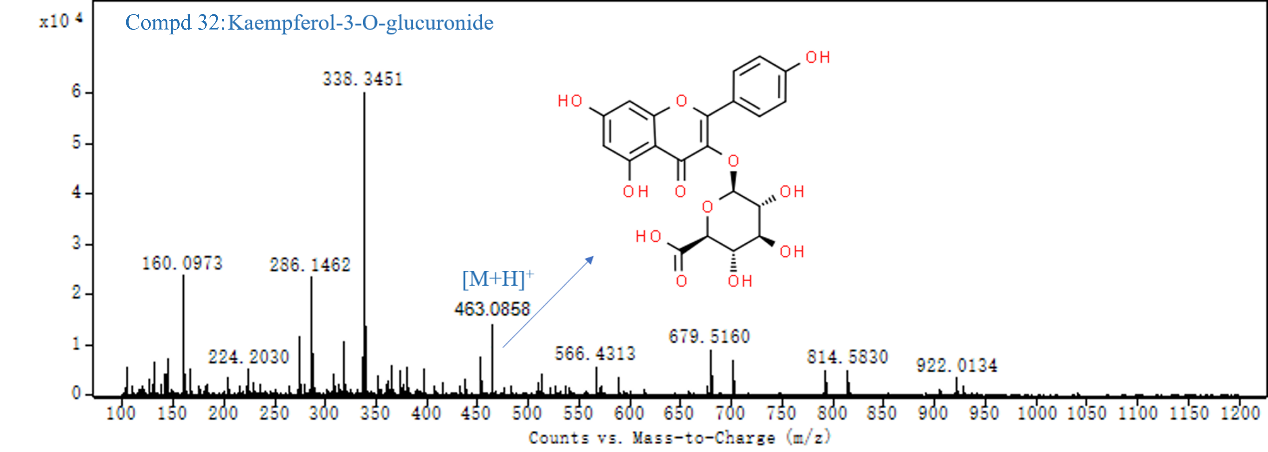


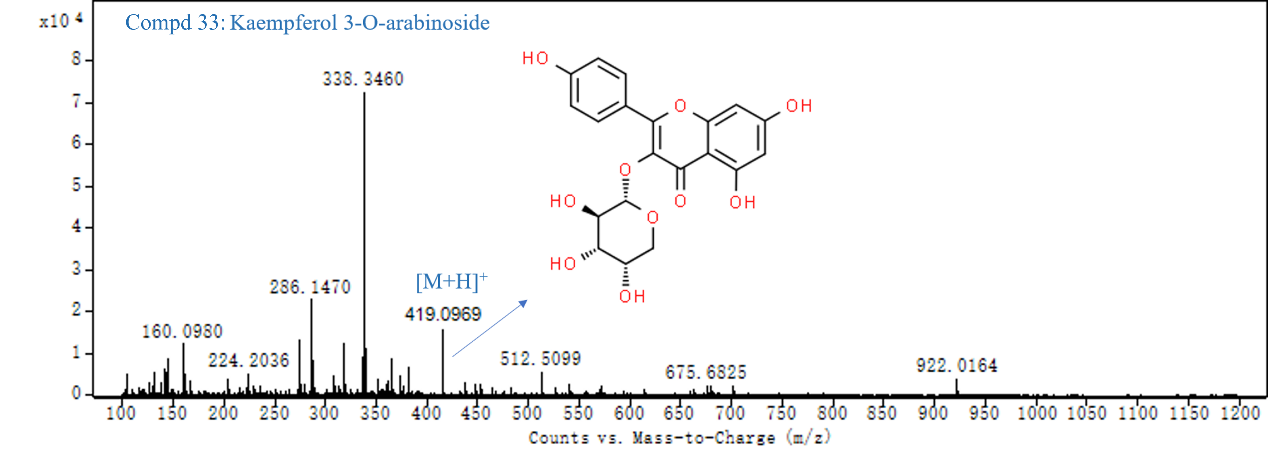


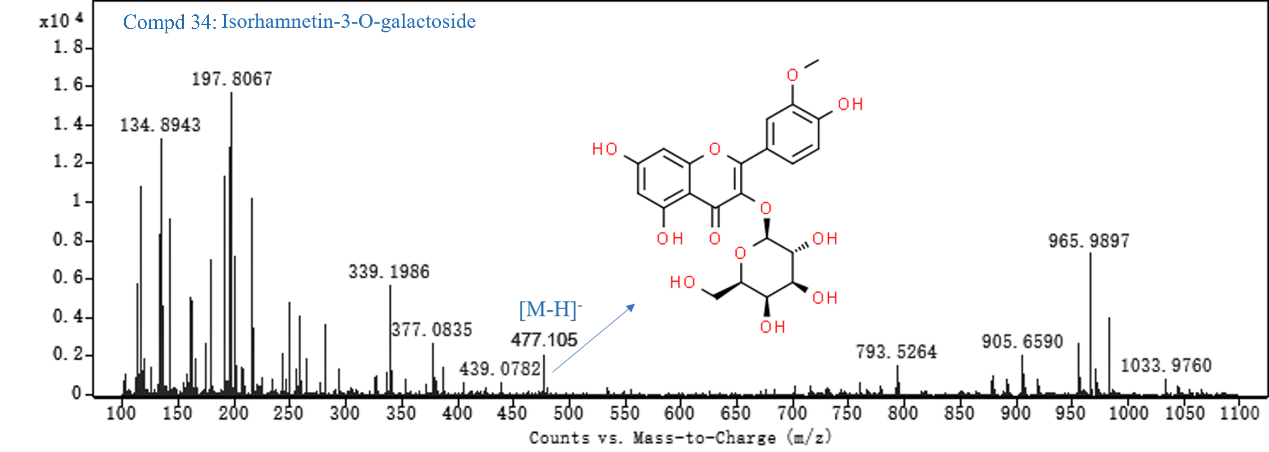


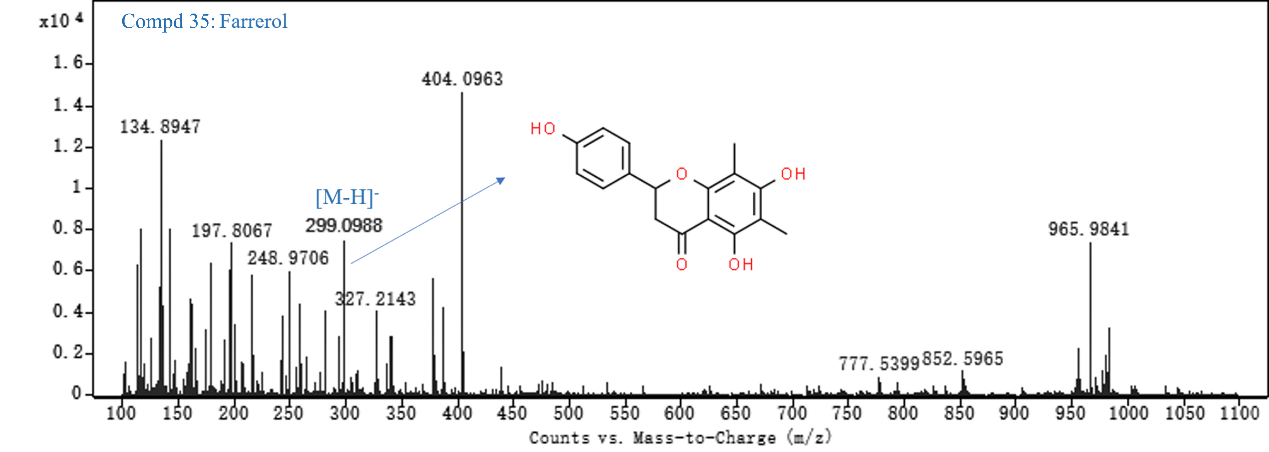


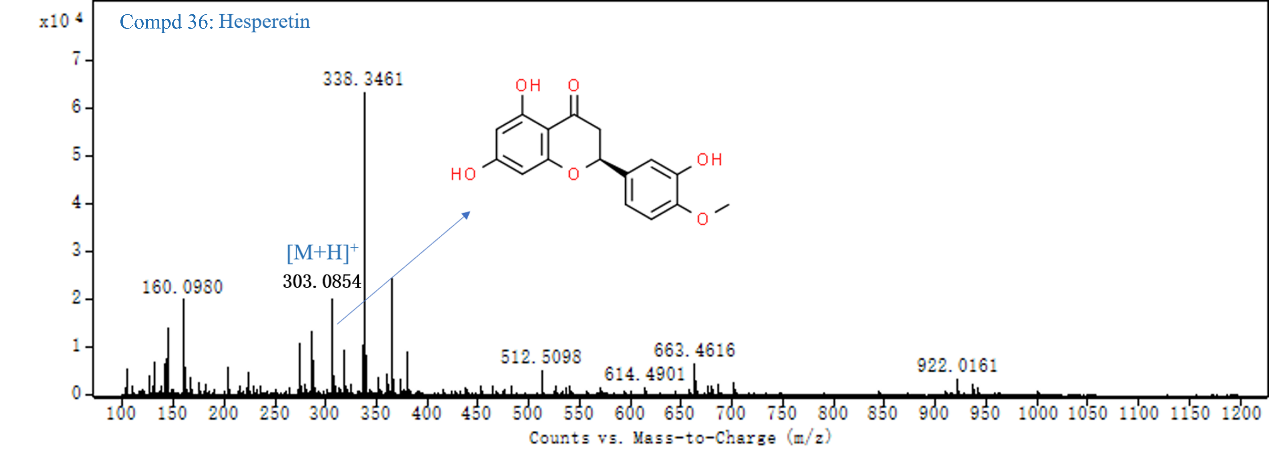


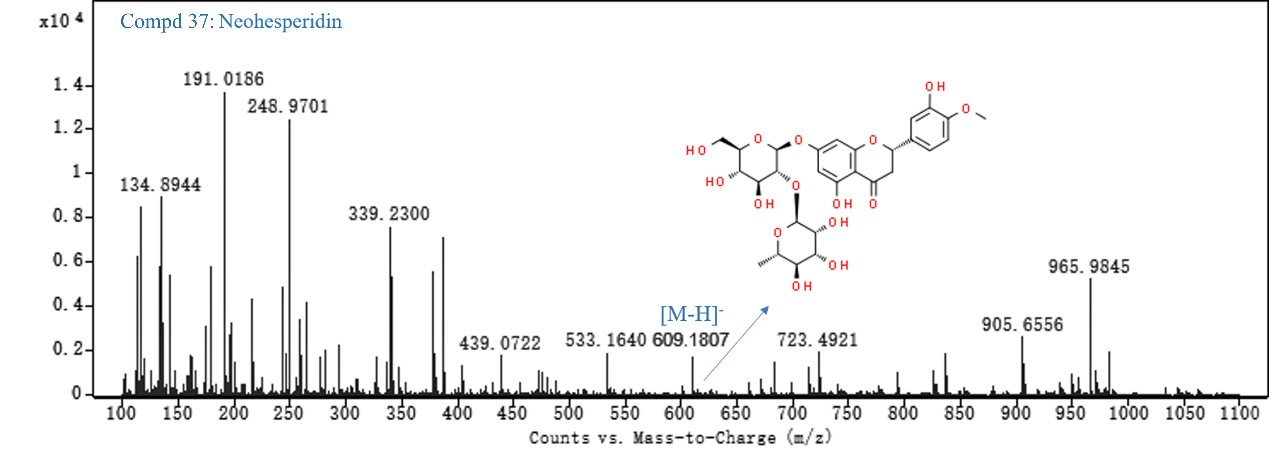


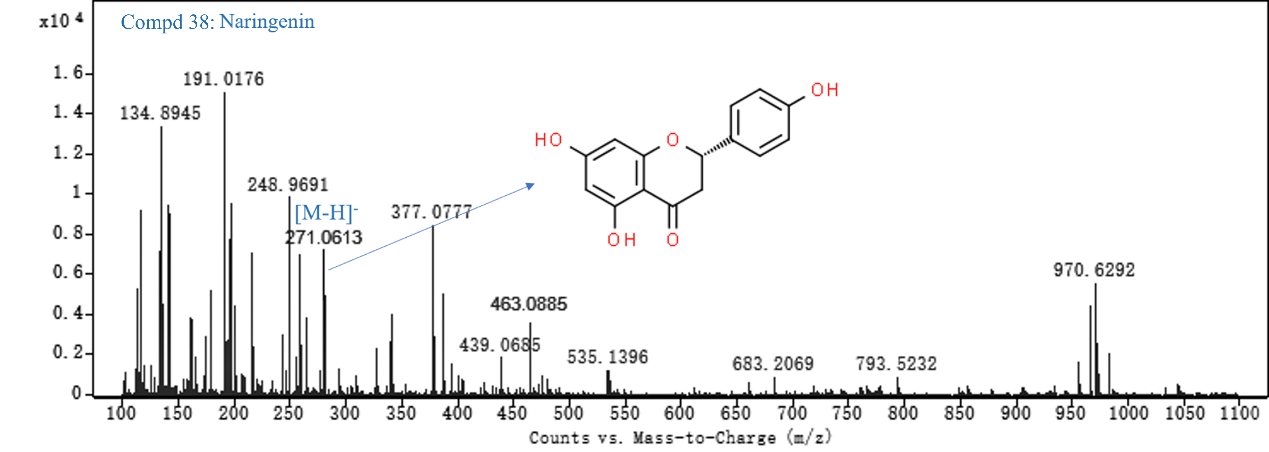


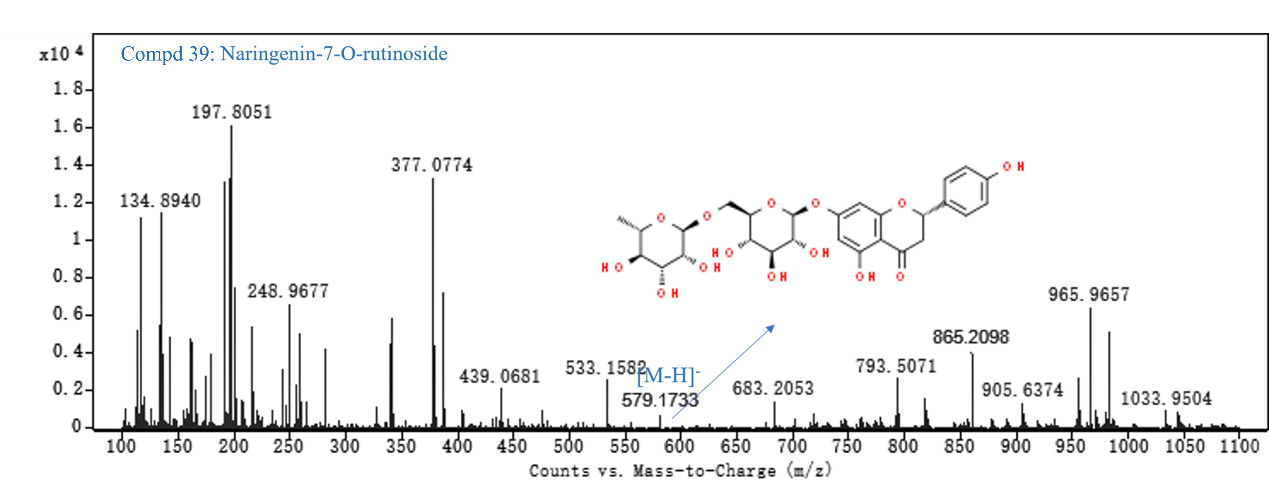


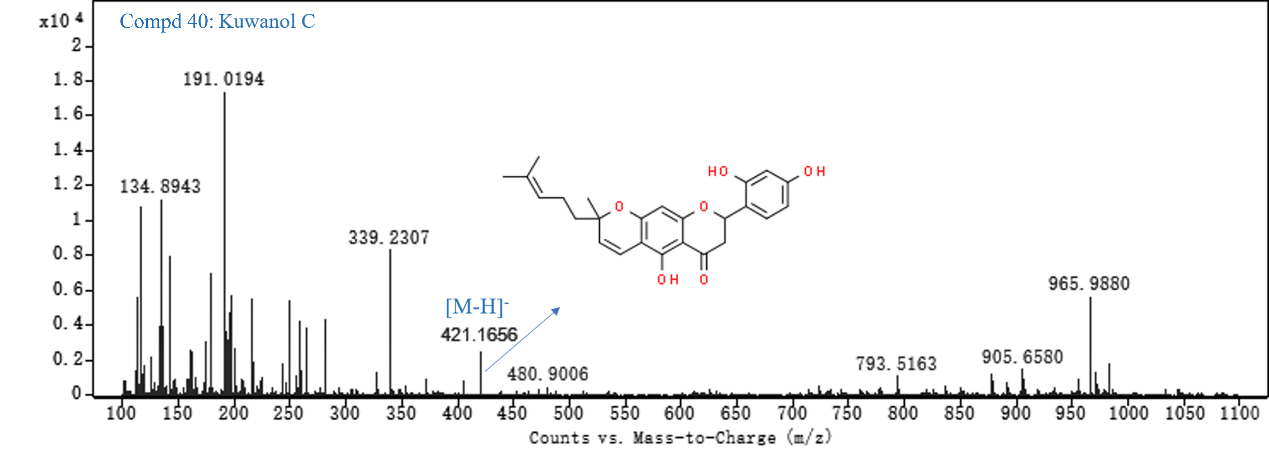


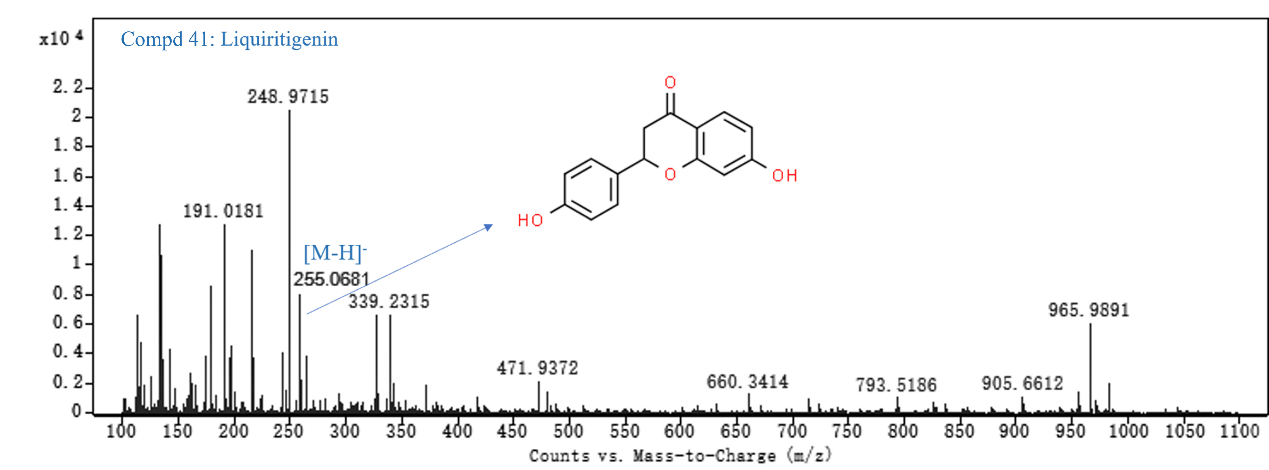


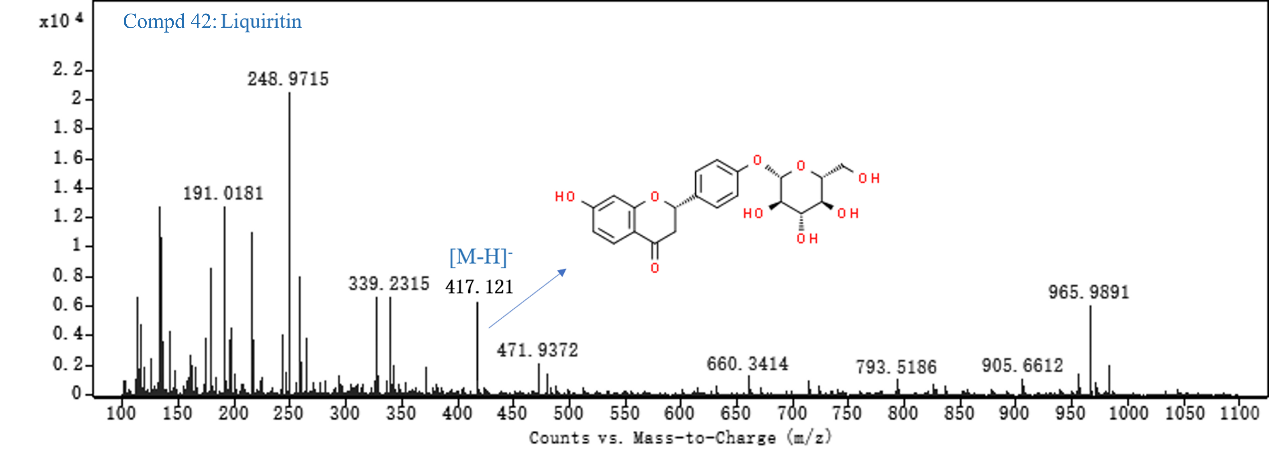


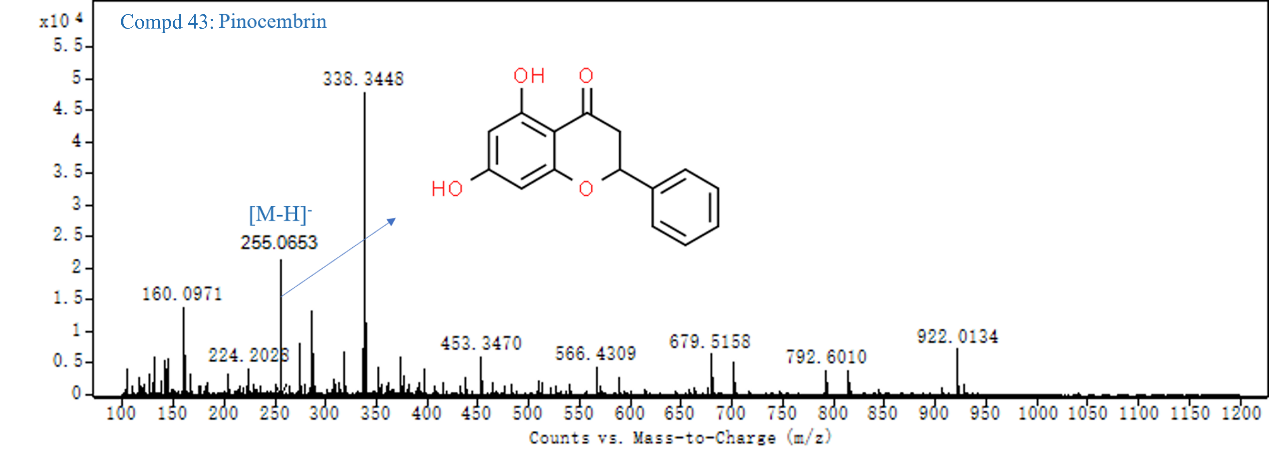


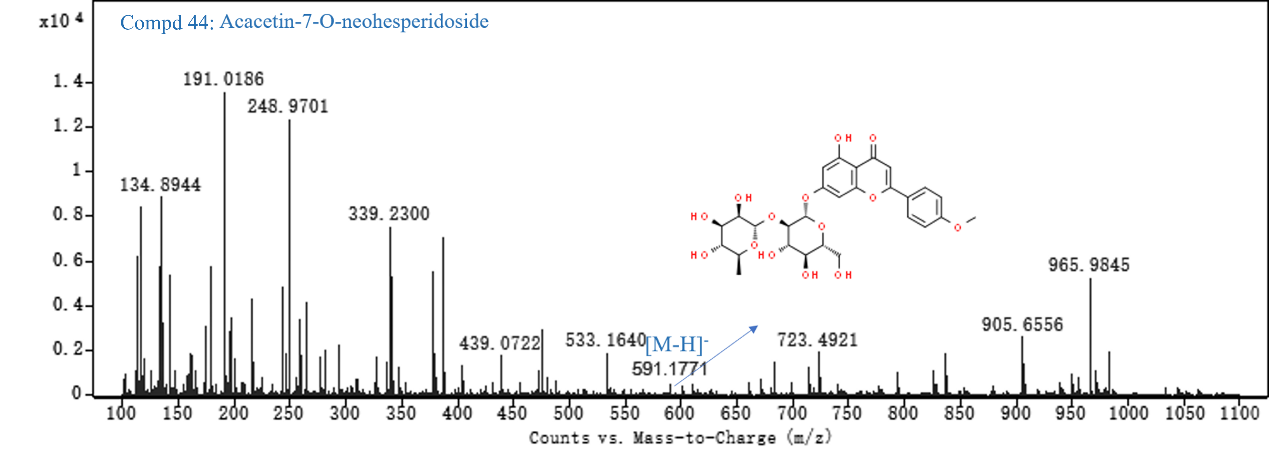


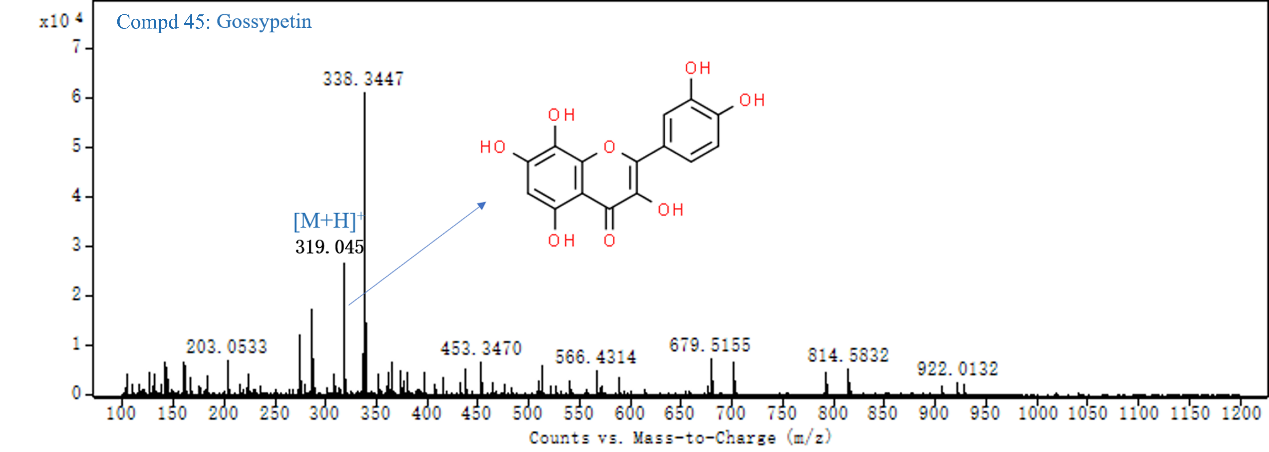


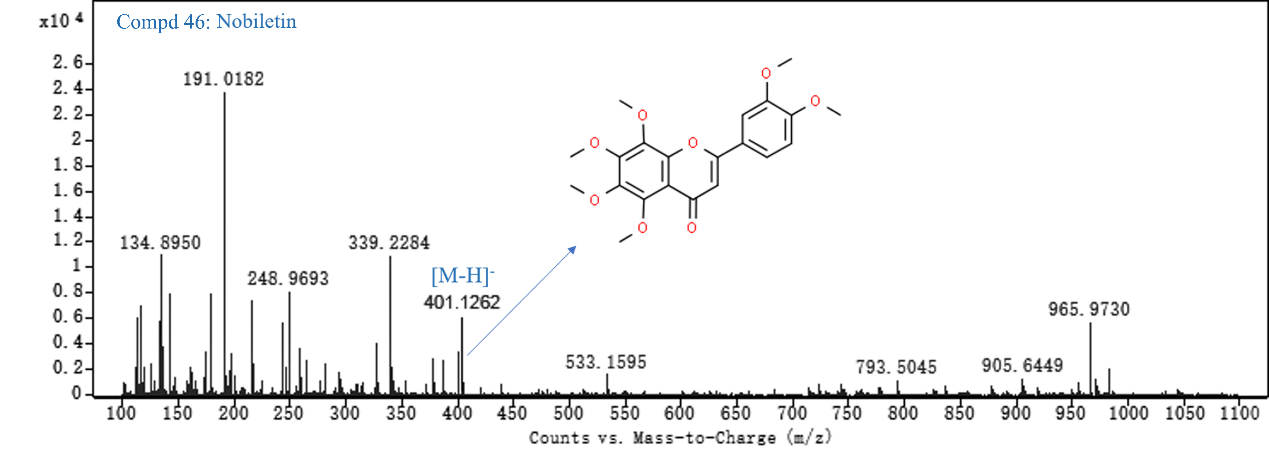


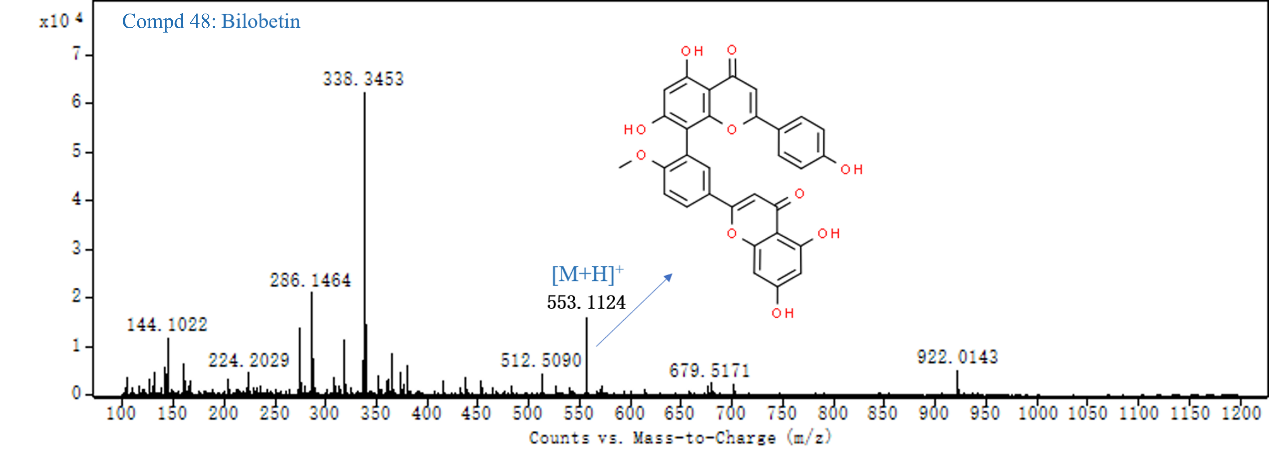


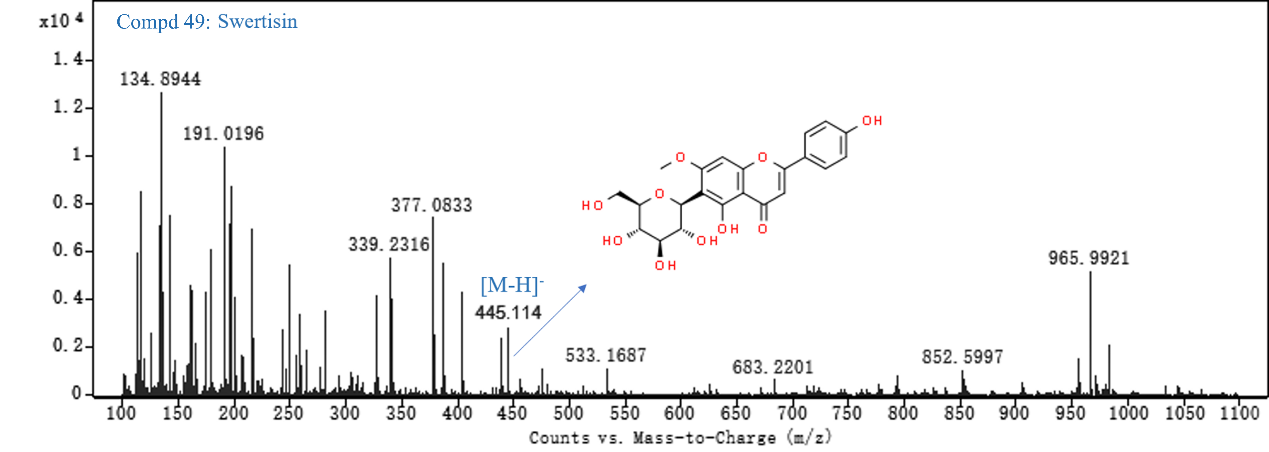


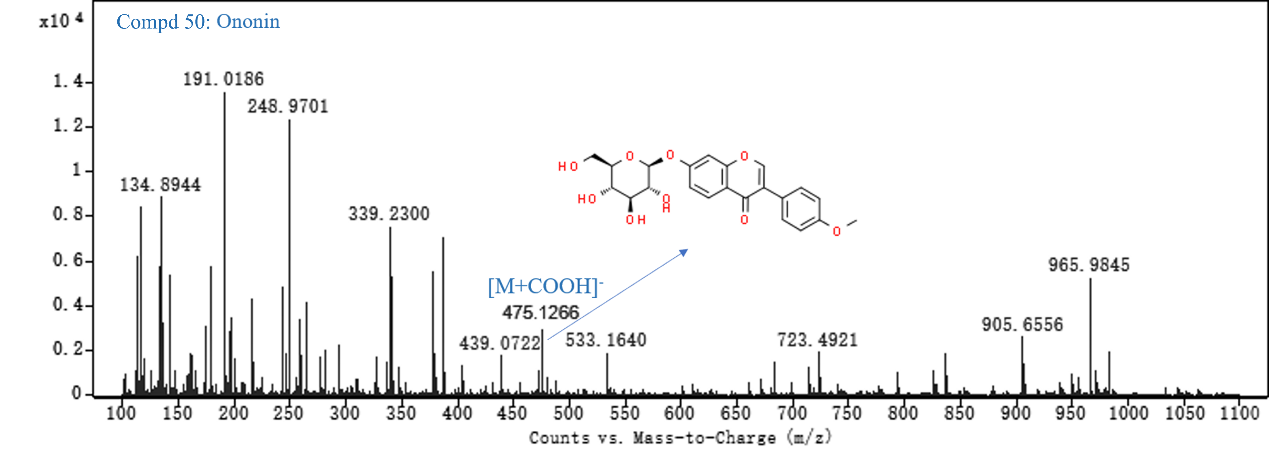


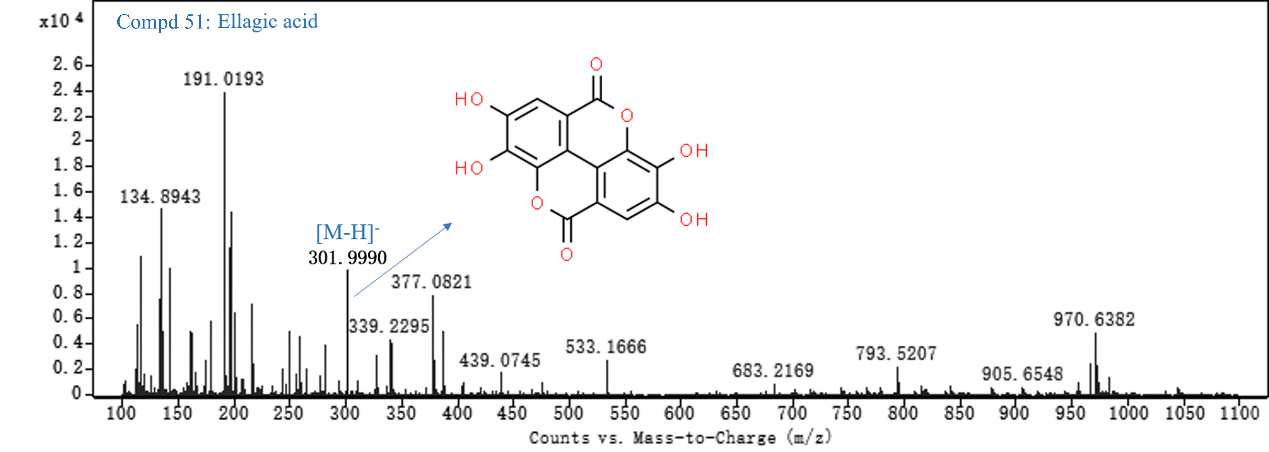


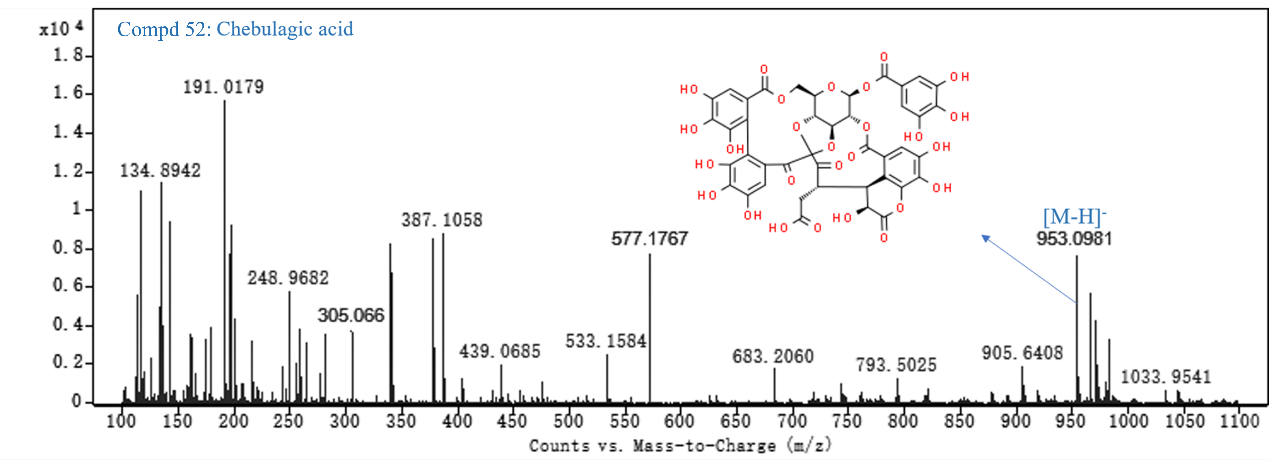


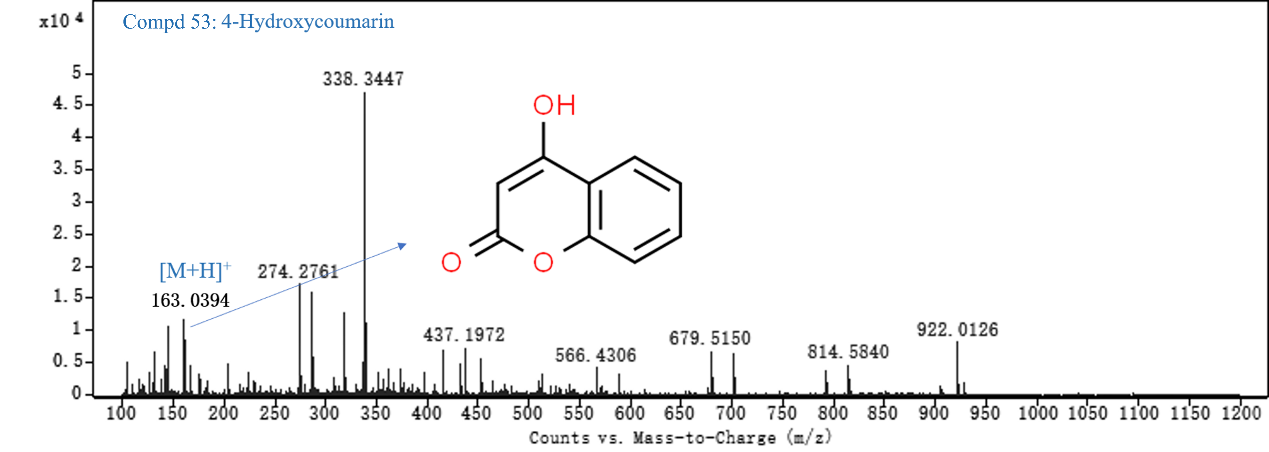


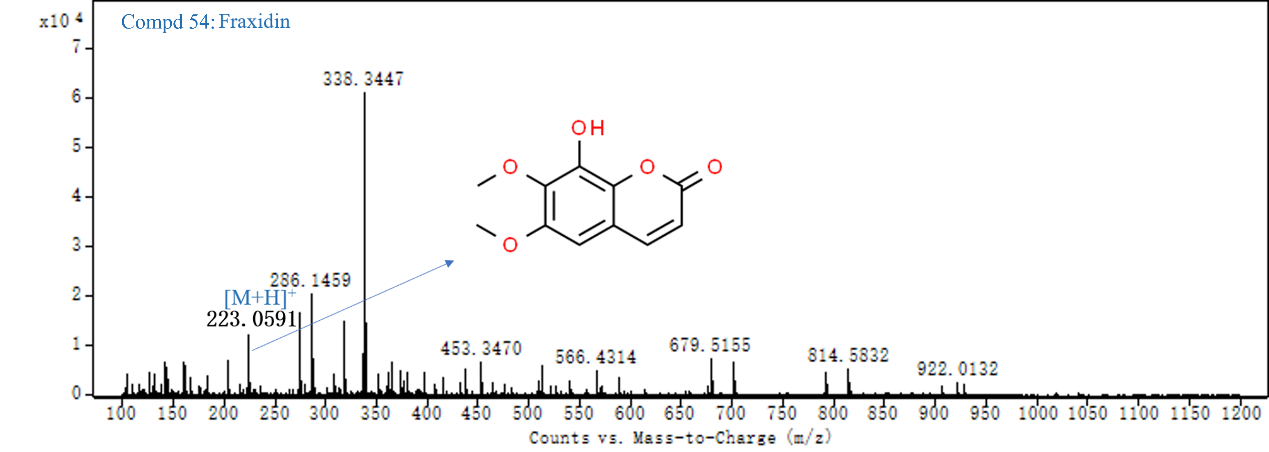


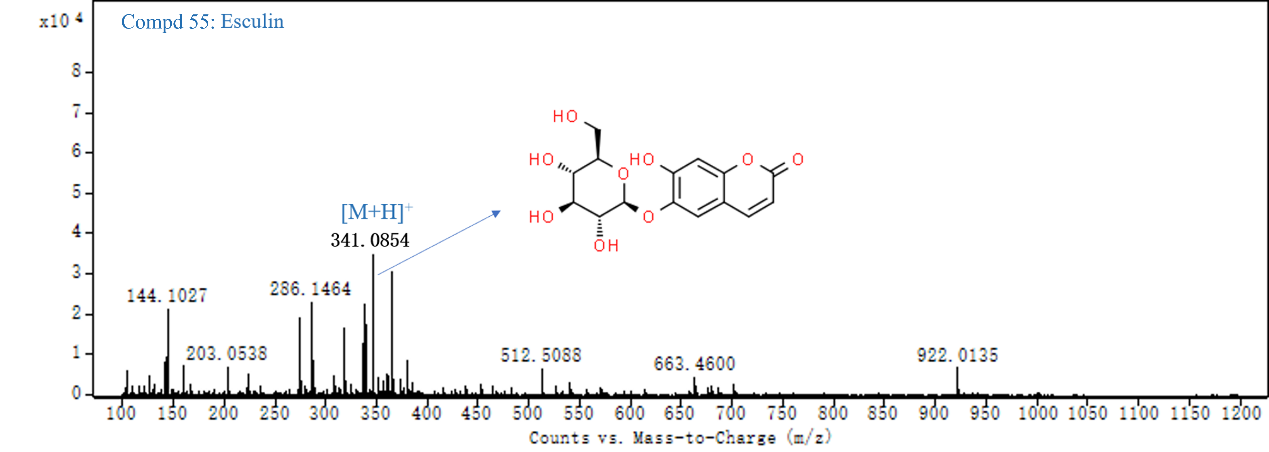


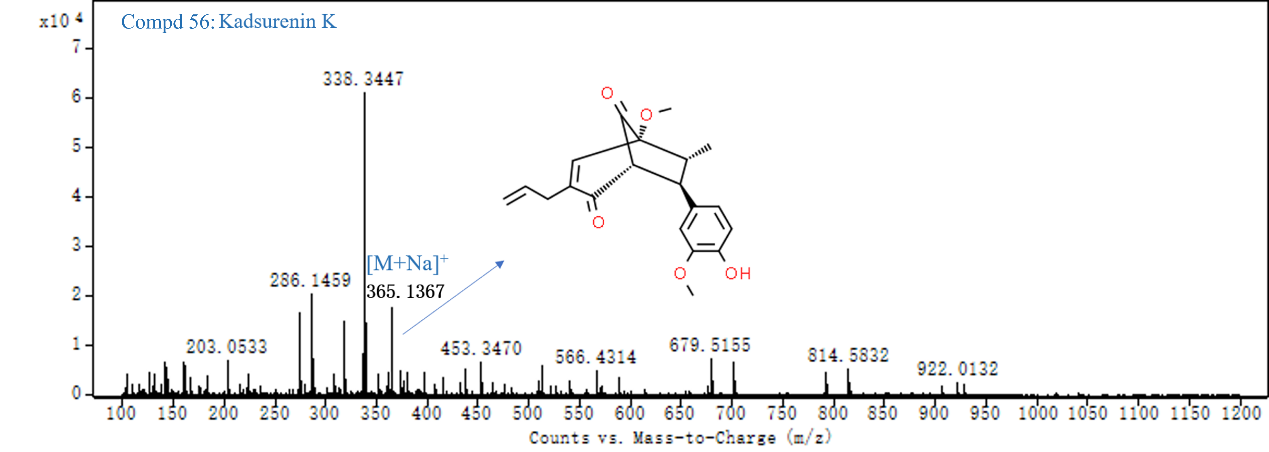


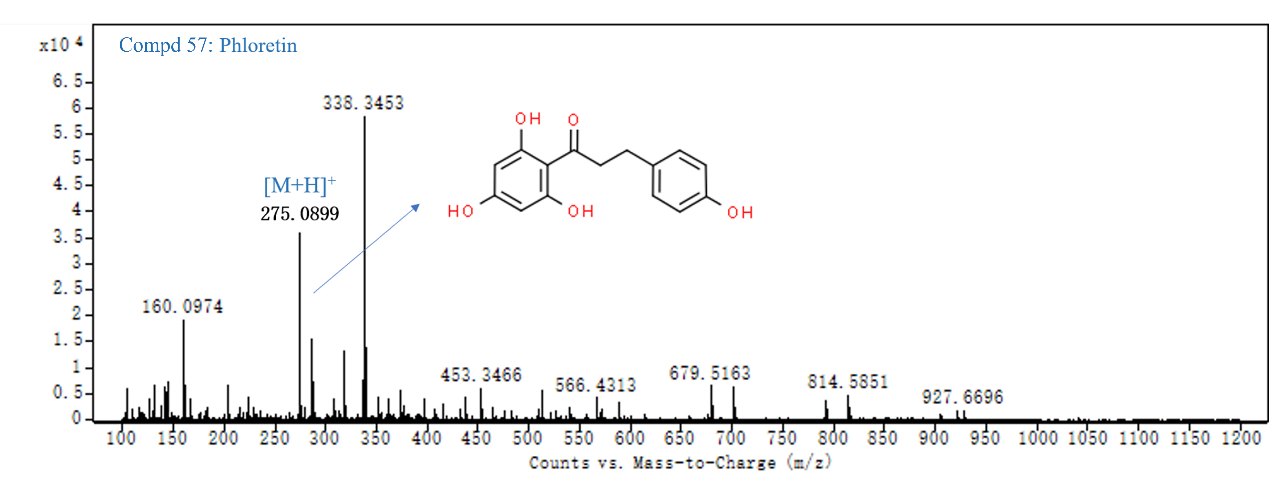


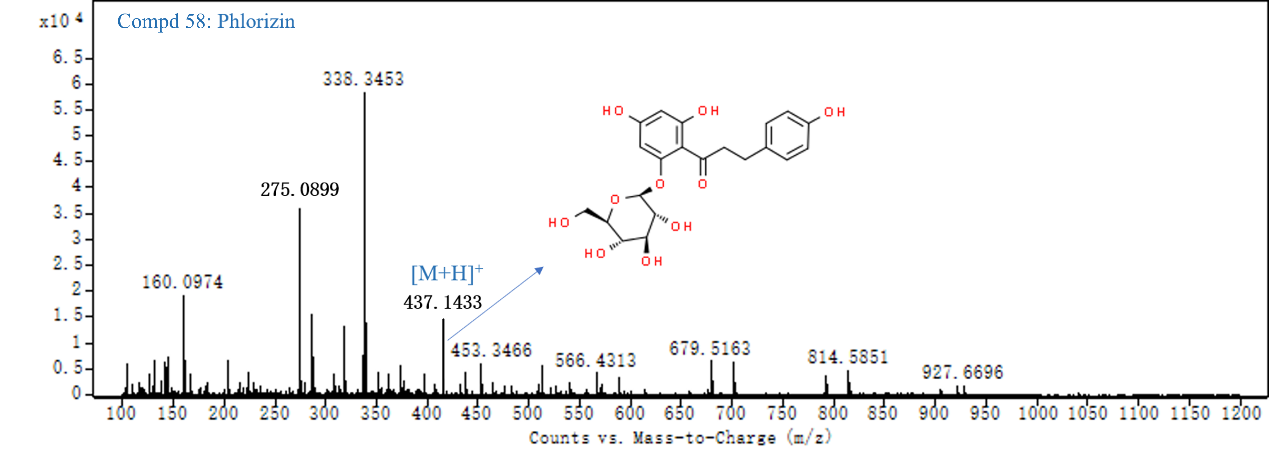


**Fig. S2.** HPLC chromatograms of 15 phenolic compounds in the mixed standards (A) and 21 jackfruit pulp samples (B).

Peaks: 1. Gallic acid; 2. Protocatechuic acid; 3. Neochlorogenic acid; 4. Procyanidin B1; 5. Catechin; 6. Chlorogenic acid; 7. Procyanidin B2; 8. Caffeic acid; 9. Epicatechin; 10. p-Coumaric acid; 11. Ferulic acid; 12. Quercitrin; 13. phlorizin; 14. Quercetin; 15. Phloretin.

**
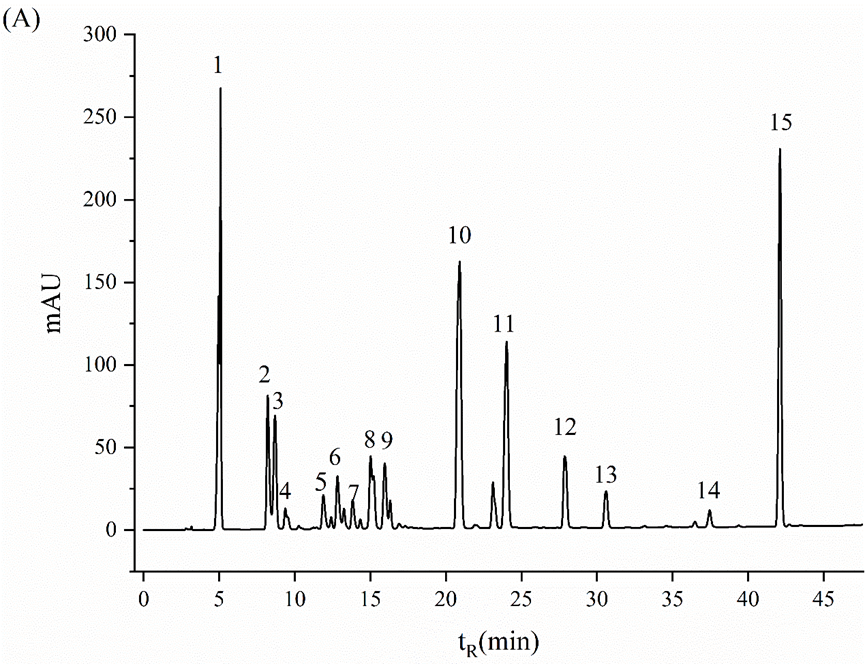
**

**
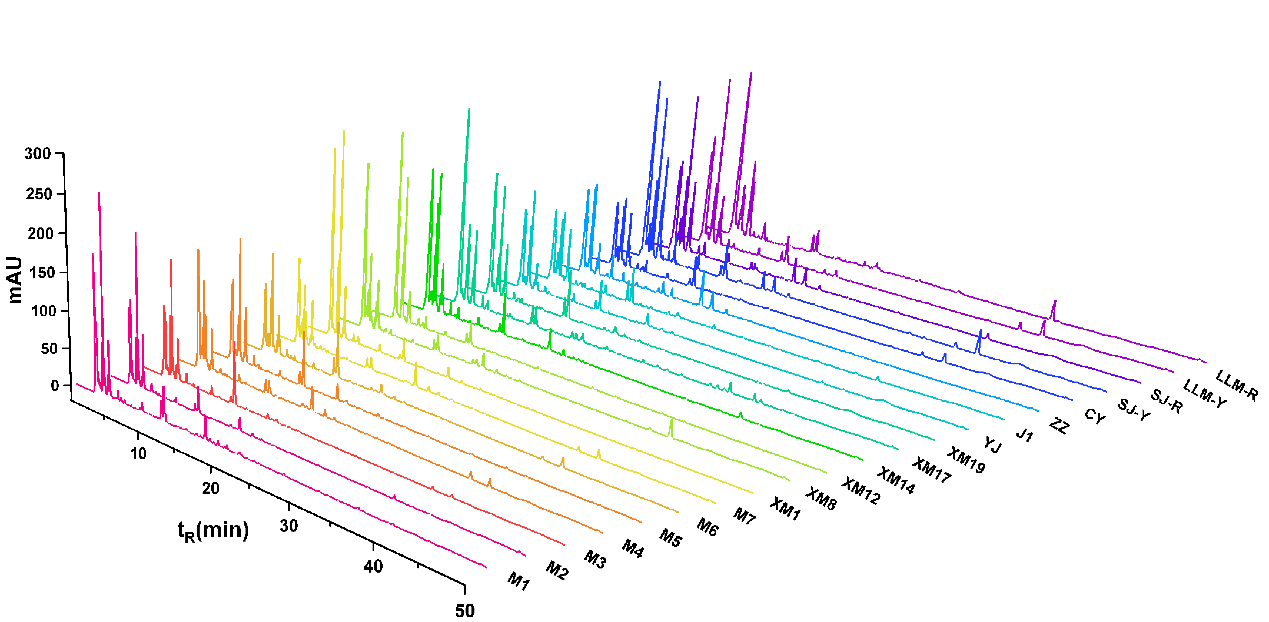
**
